# Climate-Related Distress: Latent Profiles of Eco-Anxiety, PTSD and CPTSD Symptoms and Associated Resilience and Vulnerability Factors in a Portuguese Sample

**DOI:** 10.3390/bs16101801

**Published:** 2026-10-02

**Authors:** Júlia Fonte, Amadeu Quelhas Martins, Nuno Torres, Paulo Ferrajão

**Affiliations:** 1 Faculdade de Ciências da Saúde, Universidade Europeia, 1500-210 Lisbon, Portugal; julia.fonte@ext.universidadeeuropeia.pt (J.F.); amadeu.martins@universidadeeuropeia.pt (A.Q.M.); 2 CIDESD—Research Center in Sports Sciences, Health and Human Development, 6200-001 Covilhã, Portugal; 3 UNIDCOM/IADE, Unidade de Investigação em Design e Comunicação Moscavide, 1886-502 Moscavide, Portugal; 4 William James Center, ISPA—Instituto Universitário, 1149-041 Lisboa, Portugal; ntorres@ispa.pt

**Keywords:** climate trauma, eco-anxiety, latent-profile analysis, vulnerability, resilience

## Abstract

Climate change is increasingly recognized as a significant stressor affecting well-being, yet responses to climate-related distress are heterogeneous and may reflect the interplay of eco-anxiety and trauma-related symptoms. This study used latent profile analysis to identify patterns of climate-related distress and examined their associations with environmental identity, developmental adversity, attachment, prior psychopathology, demographic characteristics, and direct exposure to climate-related events. A sample of 917 Portuguese adults completed measures of eco-anxiety, post-traumatic stress disorder (PTSD) symptom clusters, Disturbances in Self-Organization (DSO) symptoms, environmental identity, childhood adversity, and attachment. Correlates were examined using multinomial logistic regression and MANOVA. A five-profile solution was identified, ranging from minimal symptom endorsement to severe, co-occurring elevations in eco-anxiety, PTSD, and DSO symptoms. Profiles differed in Protection of Nature, but not Connectedness with Nature. Higher-distress profiles were associated with greater direct exposure to climate-related events, childhood adversity, insecure attachment, prior psychopathology, younger age, and female sex, whereas lower-distress profiles were more strongly associated with secure attachment and the absence of previous mental health difficulties. These findings support a person-centered conceptualization of climate-related distress and highlight heterogeneity in the co-occurrence of eco-anxiety and trauma-related symptoms. Climate-related distress may be embedded within developmental, psychological, demographic, and socioecological contexts.

## 1. Introduction

The contemporary world faces multiple emergencies, including conflicts, economic instability, and climate change (CC). Among these crises, CC is particularly harmful, affecting the stability of ecological and social systems and contributing to cascading impacts, from food system disruption to escalating geopolitical tensions (Scheffran, 2025; Ciullo et al., 2025). Driven predominantly by anthropogenic activity (Hogg et al., 2021), CC now affects every region of the globe, exposing populations to rising climate risks that threaten physical health and psychological well-being (Campbell-Lendrum et al., 2023; Cianconi et al., 2020).

The mental health consequences of CC can arise through multiple pathways. Individuals may be affected directly through exposure to acute weather events, such as floods, hurricanes, or wildfires, or indirectly through proximity to affected areas, witnessing environmental destruction, or exposure to information about CC through the media (Doherty & Clayton, 2011; Palinkas & Wong, 2020). These types of exposure are associated with a broad range of psychological outcomes, from transient distress to clinical conditions including depression, anxiety, insomnia, suicidal ideation, and Post-Traumatic Stress Disorder (PTSD) (Betro, 2024).

In recent years, growing attention has been given to emerging psychological concepts, including eco-emotions, which refer to emotional responses associated with awareness of environmental degradation, and psychoterratic syndromes, which describe Earth-related psychological disturbances arising from disruptions in person–place relationships (Albrecht, 2019; Hayes et al., 2018; Stanley et al., 2021; Cianconi et al., 2023). Given the breadth and heterogeneity of these responses, the present study adopts the term ‘climate-related distress’ as an umbrella construct encompassing emotional and psychological responses to actual, anticipated, or perceived climate threats. Climate-related distress may range from mild or transient discomfort to persistent and clinically significant manifestations, without implying that all such responses constitute distinct or clinically equivalent psychiatric conditions (Koder et al., 2023). This terminology is consistent with conceptual approaches that caution against pathologizing psychological responses to climate change and emphasize the importance of situating these responses within their broader social and ecological contexts (Ojala et al., 2021; Pihkala, 2020).

Eco-anxiety represents one salient manifestation of climate-related distress and has received increasing attention from scholars in recent years (Hickman, 2020). It encompasses behavioral, emotional, and cognitive responses and is predominantly future-oriented, reflecting anticipatory responses to the ongoing and anticipated consequences of the climate crisis (Clayton, 2020; Pihkala, 2020). Although eco-anxiety may arise following direct exposure to CC-related events, it more commonly reflects heightened awareness of ecological risks. At lower or manageable levels, it may function as an activating response that promotes cognitive engagement and pro-environmental behavior (Stanley et al., 2021). Conversely, when severe and accompanied by inadequate coping resources, eco-anxiety may manifest in clinically significant symptoms that interfere with daily functioning (Ferrajão et al., 2026; Jalin et al., 2024; Lutz et al., 2023).

Trauma-like responses represent another manifestation of climate-related distress and have been discussed within the broader notion of climate trauma (Cianconi et al., 2023). In the current study, climate trauma is used to refer to trauma symptomatology, occurring in the context of actual, anticipated, or cumulative climate threats, rather than as a distinct diagnostic category. Accordingly, the temporal dimension of climate trauma may encompass both past-oriented responses to experienced events and future-oriented responses to an ongoing and ever-growing threat (Kaplan, 2020; Patrick et al., 2023; Woodbury, 2019). Anticipatory distress reactions reflect intense fear and anxiety about projected catastrophes, with PTSD-like symptoms, i.e., flash-forwards, sleep disturbances and nightmares, dissociative states, and pervasive worry (Cianconi et al., 2023; Kaplan, 2020). For many individuals, climate trauma may arise following exposure to extreme weather events and life-threatening natural disasters, resulting in post-traumatic symptoms that overlap with those observed in PTSD, including heightened arousal (Nan et al., 2025; Wang & van den Berg, 2021).

As previously mentioned, PTSD is a well-documented mental health consequence of CC. Emerging evidence also suggests that symptoms of Complex PTSD (CPTSD) may occur in this context. For instance, a study conducted in a European flood-vulnerable region found that 2 of 30 flood-exposed participants met diagnostic criteria for CPTSD, while an Italian study of young people exposed to an earthquake reported prevalence rates of 9% for PTSD and 4% for CPTSD (Pedretti & Lavazza, 2026; Rossi et al., 2022). According to the ICD-11 classification, CPTSD typically develops following exposure to prolonged, repeated, or inescapable traumatic experiences, particularly those involving sustained or repeated threats (World Health Organization, 2022). In addition to the core symptom clusters of PTSD, CPTSD is characterized by persistent disturbances in self-organization (DSO), including affective dysregulation, negative self-concept, and interpersonal difficulties. Compared with PTSD, CPTSD is generally associated with greater functional impairment and higher levels of comorbid psychopathology (Brewin, 2020).

The chronic, cumulative, and pervasive nature of CC, together with the increasing frequency and severity of extreme weather events, underscores the need to examine trauma-related symptomatology. However, understanding such responses requires consideration of the individual and systemic inequalities that may shape differential vulnerability and resilience to climate-related threats (Zhang et al., 2021). A trauma and adversity model of the mental health impacts of CC conceptualizes psychological outcomes as arising from the interplay among exposure to climate-related events, cumulative adversity, pre-existing individual vulnerabilities, and protective resources (O’Donnell & Palinkas, 2024).

Within this framework, climate-related stressors may function as potentially traumatic events in themselves, particularly when exposure involves life threat, substantial disruption, or loss, while also interacting with prior developmental and/or cumulative adversity. Thus, an acute environmental shock and the activation or amplification of pre-existing vulnerabilities represent complementary pathways through which climate-related distress may emerge. Trauma-related responses to CC may therefore reactivate or exacerbate pre-existing personal, cultural, and intergenerational trauma, potentially eliciting defensive responses such as dissociation (Shomuyiwa & Lucero-Prisno, 2025; Woodbury, 2019). This perspective highlights the importance of considering both the characteristics of climate-related exposure and the developmental histories that shape individual vulnerability when examining psychological responses to CC.

### 1.1. Using Latent Profile Analysis to Identify Individual Differences in Climate-Related Distress

Framed as a continuum ranging from anticipatory to post-event impacts, climate-related distress emerges as a dynamic and heterogeneous construct shaped by individual, social, and environmental factors (Koder et al., 2023; Ojala et al., 2021; Pihkala, 2020). Such heterogeneity may not be adequately captured by traditional variable-centered approaches, which generally assume population homogeneity and focus primarily on associations among individual variables (Howard & Hoffman, 2018). In contrast, person-centered approaches, such as Latent Profile Analysis (LPA), enable the identification of distinct subgroups of individuals characterized by similar patterns of symptoms, thereby providing a more nuanced understanding of heterogeneity in climate-related distress in the context of climate change (Howard & Hoffman, 2018).

Recent studies have applied person-centered approaches to investigate climate-related distress, particularly in relation to eco-anxiety and other eco-emotions. Ágoston et al. (2024) identified five latent groups based on patterns of eco-emotions and pro-environmental behavior. Their findings indicated that greater emotional concern was associated with greater behavioral involvement, while younger adults were more likely to belong to groups characterized by moderate to high levels of anxiety, highlighting substantial heterogeneity in how individuals experience and respond to environmental concerns. Similarly, Ferrajão et al. (2026) identified five eco-anxiety profiles ranging from adaptive to highly impaired maladaptive eco-anxiety, alongside profiles characterized by psychological distress largely independent of ecological concern. Collectively, these findings suggest the utility of person-centered approaches for identifying distinct patterns of climate-related psychological responses and capturing heterogeneity that may be obscured by variable-centered analyses.

A similar person-centered perspective has been applied within the broader trauma literature to examine the latent structure of PTSD and CPTSD symptomatology across trauma-exposed populations (Bertin et al., 2025; Cloitre et al., 2013; Folke et al., 2019). These studies have identified profiles that differ not only in overall symptom severity but also in the configuration of core PTSD symptoms and DSO symptoms. For example, Teysseyre et al. (2026) identified distinct PTSD and CPTSD profiles characterized by varying levels of emotional hypoactivation, highlighting heterogeneity in affective dysregulation across trauma-related presentations. CPTSD-related profiles were more strongly associated with repeated and interpersonal trauma exposure, consistent with the ICD-11 conceptualization of CPTSD as a condition that may arise in the context of prolonged or cumulative traumatic experiences.

Evidence to date also suggests substantial heterogeneity in post-traumatic responses among populations exposed to climate-related and other environmental hazards. Using LPA in a sample of youth exposed to an earthquake, Çimen et al. (2025) identified five distinct symptom profiles: Low Symptoms, Avoidance, DSO, Mild CPTSD, and CPTSD. Their findings revealed marked variation in symptom configurations, with some profiles characterized predominantly by classical PTSD symptoms, such as avoidance and re-experiencing, whereas others showed elevated DSO symptoms, including affective dysregulation, negative self-concept, and relational difficulties. Similarly, Zhen et al. (2020) identified four distinct profiles of PTSD–depression comorbidity among flood survivors, ranging from low to severe symptom levels. More severe profiles were associated with greater trauma exposure and maladaptive emotion regulation strategies. Collectively, these findings provide further evidence for the utility of person-centered approaches in identifying distinct and multidimensional patterns of psychological responses to environmental hazards.

Building on this evidence, examining PTSD and CPTSD symptomatology alongside eco-anxiety may provide a more comprehensive characterization of climate-related distress. Accordingly, the present study uses LPA to identify distinct profiles of climate-related distress based on trauma-related symptoms corresponding to the PTSD and DSO symptom domains defined in the ICD-11, together with dimensions of eco-anxiety. This approach allows for the examination of whether distinct patterns of trauma-related symptoms and eco-anxiety co-occur within individuals, thereby providing a more nuanced understanding of the heterogeneity of psychological responses to CC.

### 1.2. Understanding Vulnerability and Resilience in Climate-Related Distress

In addition, understanding which individuals are at greatest risk for persistent mental health difficulties in the context of CC is critical for effective prevention and intervention. Risk factors identified across research on climate-related distress, particularly trauma-related responses, include demographic characteristics (e.g., female gender, younger age, lower income, and lower educational attainment), pre-event vulnerabilities such as prior psychopathology and trauma exposure, and characteristics of the disaster itself, including exposure severity and material and financial losses (Chique et al., 2021; Gauthier et al., 2025; Tang et al., 2017; Yakubu et al., 2026). Notably, a recent meta-analysis examining prospective predictors of PTSD following extreme weather events identified disaster exposure severity, lower income, and prior psychopathology as the strongest predictors of PTSD symptomatology (Gauthier et al., 2025). Complementing these findings, Yakubu et al. (2026) identified female gender, unemployment, pre-existing mental illness, repeated exposure, and severe housing damage as significant vulnerability factors for PTSD, depression, and anxiety following flood-related disasters. However, these factors are typically examined as independent predictors of specific outcomes, leaving less clear how combinations of individual vulnerabilities and exposure characteristics may correspond to distinct patterns of climate-related distress (O’Donnell & Palinkas, 2024).

Importantly, trauma-like responses to CC are not restricted to individuals directly exposed to climate-related disasters. In an Australian study, nearly 16% of participants met screening criteria for pre-traumatic stress, indicating substantial distress associated with the anticipation of future climate threats. Among individuals exposed to the impacts of CC, approximately 25% also met screening criteria for PTSD symptoms, with higher prevalence observed among women, younger adults, and socioeconomically disadvantaged individuals (Patrick et al., 2023). More broadly, economic, social, and gender inequalities may increase vulnerability to psychopathology, while climate and environmental stressors can further exacerbate existing vulnerabilities and contribute to adverse mental health outcomes (Thoma et al., 2021).

While PTSD is typically associated with exposure to one or more traumatic events, CPTSD is more commonly linked to prolonged, repeated, or cumulative traumatic experiences (Li et al., 2023). Childhood abuse is considered an important risk factor for CPTSD because it is frequently chronic, recurrent, and involves multiple forms of interpersonal victimization (e.g., sexual, physical, and emotional abuse). However, evidence also suggests that non-interpersonal traumatic experiences, including events not directly caused by another person, may be associated with CPTSD symptomatology (Hyland et al., 2017). Furthermore, polytraumatization, particularly exposure to a greater number of different traumatic experiences, has been shown to predict both PTSD and CPTSD (Cloitre et al., 2019; Kairyte et al., 2022; Karatzias et al., 2019; Redican et al., 2022). Accordingly, vulnerability to CPTSD appears to reflect not only the effects of developmental trauma but also the cumulative burden of adversity across the lifespan.

This perspective is particularly relevant to climate-related distress, as climate-related stressors may interact with pre-existing vulnerabilities and potentially precipitate or exacerbate trauma-related symptomatology. Thus, CPTSD symptom dimensions may be particularly relevant when climate-related threats intersect with prior experiences of adversity (O’Donnell & Palinkas, 2024). However, research examining CPTSD symptomatology in relation to climate-related experiences remains scarce, and evidence regarding its predictors is limited and inconsistent (Thoma et al., 2025). Addressing this gap is particularly important given the substantial functional and psychological burden associated with CPTSD, as well as emerging evidence indicating a moderate prevalence of the disorder in community samples (Hualparuca-Olivera et al., 2026).

Substantial evidence highlights the importance of identifying resilience and protective factors at both individual and community levels (Adams et al., 2021). Social support and cohesive community systems are consistently identified as protective factors against adverse mental health outcomes following disasters (Ungar & Theron, 2020), promoting self-compassion, post-traumatic growth, and prosocial behavior (Liu et al., 2021). At the individual level, resources such as adaptive coping are associated with more favorable recovery trajectories and reductions in PTSD symptoms over time (Tekin et al., 2023). Adult attachment represents another potentially important individual-level factor within this framework. Meta-analytic evidence indicates that securely attached adults report fewer PTSD symptoms, whereas insecure attachment is associated with greater symptom burden, with fearful attachment showing the strongest association with PTSD (Woodhouse et al., 2015). Consistent with these findings, secure attachment has been linked to more adaptive coping, effective emotion regulation, and greater use of social support, whereas insecure attachment is associated with difficulties in emotion regulation, reduced support-seeking, and greater vulnerability to psychological distress following adversity, including PTSD symptoms (Morison & Benight, 2022). Identifying such protective factors may therefore be particularly important for understanding why individuals exposed to similar climate-related threats exhibit markedly different patterns of psychological distress.

Additionally, a “nature buffer” effect has been documented, whereby psychological connectedness to the natural environment is associated with better mental health, including greater well-being associated with exposure to blue-green spaces and greater engagement in prosocial and pro-environmental behaviours (Clayton et al., 2021; Martin et al., 2020; White et al., 2021). However, strong connectedness to nature has also been associated with heightened vulnerability and greater emotional distress (Clayton & Karazsia, 2020; Smith et al., 2024). Environmental degradation may heighten perceptions of threat among individuals who are strongly connected to nature, while also disrupting place attachment and community resources, including social cohesion, that can contribute to resilience in a changing climate (Jennings & Bamkole, 2019; Pedretti & Lavazza, 2026; Oh et al., 2022). Taken together, these findings suggest that both individual- and community-level factors may function as either protective or vulnerability factors in the context of a changing climate.

### 1.3. Purpose of the Study and Hypotheses

Given the multifaceted pathways through which CC affects mental health, a biopsychosociocultural perspective is essential for delineating vulnerability and resilience across contexts (Shaligram et al., 2025). The heterogeneity of climate-related distress and the mechanisms that exacerbate or protect mental health at individual and community levels remain underexplored in Portugal, which is a highly vulnerable setting characterized by rising temperatures, declining precipitation, sea-level rise, and increasing frequency of extreme weather events (Carvalho et al., 2014).

Accordingly, the present study aims to characterize patterns of climate-related distress in a Portuguese sample by examining the co-occurrence of eco-anxiety and trauma-related symptoms, including PTSD symptom clusters and CPTSD-related DSO symptom dimensions. More specifically, we seek to identify distinct symptom profiles based on indicators of eco-anxiety, PTSD, and CPTSD, and to examine whether nature connectedness and protection of nature are differentially associated with these profiles. In addition, we examine whether resilience- and vulnerability-related characteristics differ across the identified profiles, thereby contributing to a more nuanced understanding of individual differences in responses to CC-related threats and experiences.

To address these aims, we employ a person-centered approach using Latent Profile Analysis (LPA) to identify subgroups characterized by different configurations and levels of climate-related distress symptoms. Based on previous person-centered research and the evidence reviewed in the Introduction, we hypothesize a four- to five-profile solution reflecting distinct patterns of co-occurring eco-anxiety and trauma-related symptoms, including the three PTSD symptom clusters and the three CPTSD-related DSO symptom dimensions. Specifically, we anticipate profiles ranging from (1) a relatively low-symptom profile, characterized by low levels of both eco-anxiety and trauma-related symptoms; (2) a low-to-moderate distress profile, characterized by modest elevations across symptom domains; (3) an elevated eco-anxiety profile, characterized by comparatively higher levels of eco-anxiety alongside lower levels of trauma-related symptoms; (4) a profile characterized by co-elevated eco-anxiety and PTSD symptoms; and (5), potentially, a profile characterized by comparatively pronounced CPTSD symptom dimensions accompanied by elevated eco-anxiety. These profiles are conceptualized as empirically derived patterns of symptom endorsement rather than distinct clinical or diagnostic categories.

We further hypothesize that nature connectedness and protection of nature will show differential associations with the identified symptom profiles. Given the potentially dual role of engagement with the natural environment, we expect these characteristics to vary across profiles characterized by different configurations and levels of eco-anxiety and trauma-related symptoms. Specifically, profiles characterized by lower overall symptom levels are expected to exhibit comparatively higher levels of nature connectedness and protection of nature, whereas profiles characterized by greater eco-anxiety may also show elevated levels of these characteristics, consistent with the possibility that stronger engagement with and concern for the natural environment co-occur with heightened awareness of environmental degradation and climate-related threats. Thus, we expect nature connectedness and protection of nature to differ across symptom profiles, without presuming a uniform direction or causal role in relation to climate-related distress.

Finally, we hypothesize that resilience- and vulnerability-related characteristics will differ across the identified symptom profiles. Specifically, characteristics conceptually related to greater resilience are expected to be more strongly represented in profiles characterized by lower levels of climate-related distress, whereas vulnerability-related characteristics are expected to be more strongly represented in profiles characterized by higher or more co-occurring levels of eco-anxiety and trauma-related symptoms. These hypotheses concern cross-sectional differences in the distribution of these characteristics across empirically derived symptom profiles and are intended to characterize patterns of association rather than to imply that such characteristics constitute causal risk or protective factors, or that they prospectively predict membership in any particular profile. Accordingly, the identified profiles should be interpreted as descriptive configurations of symptom levels and associated characteristics rather than as distinct clinical, diagnostic, vulnerability, or resilience groups.

## 2. Materials and Methods

### 2.1. Sample and Study Design

The sample consisted of 917 native Portuguese adults aged 18 years and older (M = 35.00, SD = 16.13; range = 18–83). As shown in Table 1, the sample was predominantly female (63%) and single (55%). Regarding educational level, the majority of participants reported having completed at least 12 years of formal education, with a considerable proportion holding a university degree. In contrast, only a small percentage reported nine or fewer years of schooling. With respect to occupational status, most participants were either employed or students. Socioeconomic status was predominantly self-reported as middle class, followed by smaller proportions indicating upper-middle or low status, whereas only a minimal fraction reported belonging to a high socioeconomic stratum (see Table 1).

This study forms part of a broader research project examining psychological variables associated with climate change. The main objective was to assess climate change-related concerns and related emotional response variables in a sample of Portuguese adults. Ethical approval for the study protocol was granted by the Institutional Review Board of Universidade Europeia (ethics approval reference number PI23PF) on 17 July 2024. Data were collected using a convenience sampling approach, with participants recruited through mailing lists and social media platforms (Facebook, Instagram, LinkedIn, and WhatsApp).

Inclusion criteria required participants to be Portuguese nationals and aged 18 years or older. Prior to participation, individuals were informed about the aims and procedures of the study in accordance with the Declaration of Helsinki, and electronic informed consent was obtained from all participants. Participation was voluntary and unpaid. Data collection was conducted via an online survey consisting of self-report measures assessing eco-anxiety, environmental identity, psychological symptoms, and sociodemographic characteristics. The survey was conducted through a web-based platform and required approximately 20 min to be completed. No missing data were present in the dataset, as the survey was programmed to require completion of all study measures prior to submission. Consequently, only participants who completed the full set of measures were able to submit the questionnaire and be included in the analyses.

### 2.2. Measures

#### 2.2.1. International Trauma Questionnaire

The International Trauma Questionnaire (ITQ; Cloitre et al., 2018; Rocha et al., 2022) is an 18-item self-report instrument developed to assess PTSD and CPTSD according to the ICD-11. PTSD is represented by three symptom clusters—re-experiencing, avoidance, and sense of threat—whereas CPTSD comprises these PTSD clusters plus three DSO dimensions: affective dysregulation, negative self-concept, and disturbances in relationships. Items are rated on a 5-point Likert scale (0 = “Not at all” to 4 = “Extremely”) and assess six symptom clusters: re-experiencing, avoidance, and sense of threat, corresponding to PTSD, and affective dysregulation, negative self-concept, and disturbances in relationships, corresponding to the disturbances in self-organization (DSO) component of CPTSD. In the present study, the ITQ was adapted to examine PTSD- and CPTSD-like symptomatology in the context of climate-related distress. Specifically, the wording of the items was modified to refer to both experiences and anticipated threats associated with climate change. This adaptation was theoretically informed by the chronic, cumulative, and pervasive nature of climate change, which may involve both exposure to actual climate-related events and persistent anticipation of future climate-related threats. Accordingly, climate-related distress was conceptualised as encompassing responses to climate-related experiences as well as perceived or anticipated threats, rather than being restricted to responses to a discrete past traumatic event. Given that the adapted instrument has not been independently validated as a measure of ICD-11 PTSD or CPTSD, the present study does not interpret the resulting scores as establishing PTSD or CPTSD diagnoses. Rather, the ICD-11 diagnostic algorithms were applied as an exploratory and theoretically anchored framework to examine whether participants’ responses to climate-related stressors exhibited symptom patterns broadly consistent with the PTSD and CPTSD constructs. Thus, the resulting classifications should be understood as indicative of PTSD- and CPTSD-like symptomatology in the context of climate-related distress, rather than as clinical diagnoses. Functional impairment criteria were retained in this exploratory application to preserve the conceptual distinction between symptom endorsement and clinically meaningful interference. Subscale scores were calculated by summing the items within each symptom cluster, with higher scores indicating greater symptom severity. The adapted ITQ was administered to participants in this form.

To further examine the psychometric properties of the adapted measure, a confirmatory factor analysis (CFA) was conducted using IBM SPSS Amos (version 29; IBM Corp., Armonk, NY, USA). Consistent with the factor structure proposed in previous psychometric research on the ITQ, the model comprised six symptom dimensions: Re-experiencing, Avoidance, and Sense of Threat for PTSD, and Affect Dysregulation, Negative Self-Concept, and Disturbances in Relationships for CPTSD. The ITQ symptom items were specified as observed variables loading onto their respective latent factors, and the model was estimated using the Maximum Likelihood method. Although the ITQ items are ordinal, they were treated as continuous variables for the purposes of Maximum Likelihood estimation, consistent with the analytic approach adopted in this study. In accordance with previous psychometric applications of the ITQ, the functional-impairment items were not included in the factor model, as these items assess the functional consequences of PTSD and CPTSD symptomatology rather than representing indicators of the underlying symptom dimensions. Model fit was evaluated using conventional criteria: non-significant χ^2^; χ^2^/df ratio between 1 and 5; Normed Fit Index (NFI), Comparative Fit Index (CFI), and Tucker–Lewis Index (TLI) greater than 0.95; and Root Mean Square Error of Approximation (RMSEA) and Standardized Root Mean Square Residual (SRMR) values between 0.00 and 0.06 (Kline, 2016). Factor loadings exceeding 0.30 were considered acceptable (Hair et al., 2010). The model showed a good fit to the data, χ^2^(134) = 157.65, *p* = 0.08, χ^2^/df = 1.18, NFI = 0.99, CFI = 0.99, TLI = 0.99, RMSEA = 0.01, and SRMR = 0.02. Standardized factor loadings were examined to assess the strength of the associations between the observed symptom items and their respective latent factors (Table 2). Internal consistency was also good, with Cronbach’s α = 0.88 for the PTSD symptom clusters and α = 0.91 for the CPTSD symptom clusters. These findings provide preliminary evidence of the structural and internal consistency of the adapted measure in this sample; however, they should not be interpreted as constituting full validation of the adapted ITQ for the assessment or diagnosis of ICD-11 PTSD or CPTSD in the context of climate change.

#### 2.2.2. Hogg Eco-Anxiety Scale

The Hogg Eco-Anxiety Scale (HEAS; Hogg et al., 2021; Ferrajão et al., 2025) is a 13-item self-report instrument used to assess eco-anxiety symptoms experienced over the previous two weeks. Responses are provided on a 5-point Likert scale ranging from 0 (“not at all”) to 4 (“every day”). The scale comprises four dimensions: emotional symptoms (e.g., feeling nervous, anxious, or on edge), rumination (e.g., difficulty disengaging from thoughts about future climate change and broader environmental problems), behavioral symptoms (e.g., difficulties in working and/or studying), and anxiety related to personal impact (e.g., concerns about one’s responsibility in addressing environmental issues). In accordance with the original structure of the HEAS, none of the items are reverse-worded; therefore, no reverse coding was required during the scoring procedure. Dimension scores were computed by summing the items within each respective subscale, with higher scores indicating greater eco-anxiety. Internal consistency was good across subscales: emotional symptoms (α = 0.85), rumination (α = 0.85), behavioral symptoms (α = 0.81), and anxiety about personal impact (α = 0.86).

#### 2.2.3. Childhood Trauma Questionnaire

The Childhood Trauma Questionnaire (CTQ; D. P. Bernstein et al., 2003; Dias et al., 2013) is a 28-item retrospective self-report measure designed to assess experiences of maltreatment during childhood and adolescence. It evaluates five domains of early adverse experiences: emotional abuse, physical abuse, sexual abuse, emotional neglect, and physical neglect. Items are rated on a 5-point Likert scale ranging from 1 (“never true”) to 5 (“very often true”). Subscale scores are calculated by summing the items within each domain, with higher scores indicating greater severity of childhood trauma exposure. Established cut-off values were used to classify moderate and severe levels of maltreatment across subscales, following D. Bernstein and Fink (1998). The CTQ demonstrated satisfactory to good internal consistency in the present study, with Cronbach’s alpha coefficients of 0.86 (emotional abuse), 0.84 (physical abuse), 0.91 (sexual abuse), 0.85 (emotional neglect), and 0.73 (physical neglect).

#### 2.2.4. Revised Adult Attachment Scale

The Revised Adult Attachment Scale (RAAS; Collins & Read, 1990; Canavarro, 1997) is an 18-item self-report questionnaire that employs a five-point Likert scale (1 = Not at all characteristic, 5 = Very characteristic) to evaluate three dimensions of adult attachment: closeness (e.g., “I find it relatively easy to get close to others”), dependence (e.g., “I am comfortable depending on others”), and anxiety (e.g., “I often worry that my partner does not really love me”). Attachment anxiety was indexed by the sum of items on the anxious dimension, and attachment avoidance was indexed by the sum of the closeness and dependency dimensions. Categorical attachment styles were defined by participants’ scores on these dimensions: secure (low anxiety, low avoidance), preoccupied (high anxiety, low avoidance), dismissing (low anxiety, high avoidance), and fearful (high anxiety, high avoidance). Specifically, scores above the midpoint of the 5-point response scale were classified as high, whereas scores below the midpoint were classified as low; scores at the midpoint were not assigned to a categorical attachment style. This categorization reflects the original RAAS scoring procedure (Collins, 1996; Collins & Read, 1990) rather than a cut-off established specifically for the present study. The scale demonstrated satisfactory internal consistency, with Cronbach’s alpha coefficients of 0.78 for closeness, 0.75 for dependence, and 0.88 for anxiety.

#### 2.2.5. Revised Environmental Identity Scale

The Revised Environmental Identity Scale (Revised EID; Clayton et al., 2021; Ferrajão et al., 2024) is a 14-item self-report instrument used to assess environmental identity. Items are rated on a 7-point Likert scale ranging from 1 (“not at all”) to 7 (“true of me”). Although initially proposed as a unidimensional measure, cross-cultural findings, including the Portuguese validation, support a two-factor structure comprising Connectedness with Nature (e.g., “I think of myself as a part of nature, not separate from it”) and Protection of Nature (e.g., “I consider myself a steward of our natural resources”). The item examples provided for each dimension are illustrative only and are intended to clarify the content represented by the respective factors, rather than to provide an exhaustive or systematic presentation of scale items. Scores for each dimension were computed by summing the responses to the items comprising the respective dimension, with higher scores indicating a stronger environmental identity. The scale demonstrated good internal consistency, with Cronbach’s alpha coefficients of 0.94 for Connectedness with Nature and 0.81 for Protection of Nature.

#### 2.2.6. Climate Change Exposure and Psychiatric History

Information was additionally collected on direct exposure to climate change–related events (e.g., storms, floods, droughts, and wildfires) and on the presence of prior psychiatric conditions. All variables were assessed using dichotomous self-report questions (yes/no).

#### 2.2.7. Sociodemographic Variables

Sociodemographic information was collected, including participants’ gender, age, marital status, educational attainment, occupational status, residential setting, and socioeconomic background.

### 2.3. Statistical Methods

Latent profile analysis (LPA) was conducted in R (version 4.0) using the tidyLPA package with Jamovi (Version 28.3) to identify distinct patterns of climate-related distress. The model included six trauma-related indicators corresponding to the three ICD-11 PTSD symptom clusters (re-experiencing, avoidance, and sense of threat) and the three CPTSD-related disturbances in DSO dimensions (affective dysregulation, negative self-concept, and disturbances in relationships), along with four eco-anxiety dimensions, all specified as continuous indicators using standardized (z-score) values. Models were estimated within a Gaussian finite mixture framework, assuming conditional independence of indicators within profiles and class-invariant variances with zero covariances (i.e., diagonal variance–covariance matrices). Robust maximum likelihood estimation was applied, with multiple random starts (e.g., 1000 initial and 250 final optimizations) to minimize the likelihood of local solutions. Solutions specifying two to eight latent profiles were examined.

Model selection was based on a combination of statistical fit indices and substantive considerations. These included the Akaike Information Criterion (AIC), Bayesian Information Criterion (BIC), and sample-size adjusted BIC (SSABIC), with lower values indicating better model fit and parsimony (Lanza et al., 2013). The Bootstrap Likelihood Ratio Test (BLRT) was used to compare nested models, with non-significant values (*p* > 0.05) favoring the more parsimonious solution. Entropy was used to assess classification accuracy, with higher values reflecting clearer delineation between profiles. In line with prior recommendations, entropy values of 0.40, 0.60, and 0.80 were interpreted as indicating low, moderate, and high classification precision, respectively. Solutions in which the smallest class comprised less than 5% of the sample were interpreted cautiously and evaluated alongside statistical fit indices and theoretical interpretability (Lo et al., 2001; Masyn, 2013).

Subsequent analyses were performed using IBM SPSS Statistics (Version 29). A multivariate analysis of variance (MANOVA) was conducted to assess differences among the latent profiles with respect to Connectedness with Nature and Protection of Nature. Pairwise comparisons were examined with Bonferroni-adjusted post hoc tests to control for the familywise error rate. These procedures were intended to identify patterns of resilience and vulnerability associated with Connectedness with Nature and Protection of Nature across the profiles derived from the LPA.

Multinomial logistic regression analyses examined associations between latent class membership (based on posterior probabilities assigning individuals to their most likely class) and sociodemographic, exposure, and clinical variables, including gender, age, marital status, educational attainment, professional and socioeconomic status, direct exposure to storms, floods, droughts, and wildfires, and prior psychiatric conditions (all coded dichotomously). Given the absence of a universally applicable minimum sample-size criterion for multinomial logistic regression, model adequacy was evaluated based on the observed sample size, the number of predictors, and the distribution of observations across outcome categories. Model fit was assessed using Nagelkerke R^2^ (Nagelkerke, 1991), a rescaled version of Cox–Snell R^2^ (Cox & Snell, 1989), and odds ratios were interpreted as the relative change in likelihood of class membership compared with the reference group.

## 3. Results

### 3.1. Latent Profile Analysis

The optimal latent profile solution was determined by jointly considering statistical fit, likelihood-ratio tests, classification accuracy, profile size, parsimony, and substantive interpretability (Lanza et al., 2013; Masyn, 2013). As shown in Table 3, AIC, BIC, and SSABIC continued to decrease, and likelihood-ratio tests remained significant as additional profiles were added, indicating incremental improvements in statistical fit with increasing model complexity. Thus, the five-profile solution was not selected on the basis of statistical fit indices alone. Rather, consideration of profile size, parsimony, classification quality, and substantive interpretability favored the five-profile solution. The five-profile solution was the last solution in which all profiles comprised a substantively adequate proportion of the sample, with the smallest profile representing 5.2% of participants. In contrast, the six-profile solution introduced a substantially smaller profile (3.1%), with similarly small profiles emerging in the seven- and eight-profile solutions. Although no universal minimum profile-size criterion exists, increasingly small profiles may be more susceptible to sampling variability and may indicate over-extraction of additional heterogeneity. Moreover, the additional profiles in the six- to eight-profile solutions did not provide sufficient substantive differentiation to outweigh the loss of parsimony associated with their inclusion. Accordingly, the five-profile solution was retained as the most parsimonious and substantively interpretable representation of the data, providing a balance between statistical fit, classification quality, profile-size adequacy, and interpretability.

To further examine the robustness of the latent profile solution to the covariance structure imposed in the primary LPA, an alternative model allowing profile-specific variances across the ten indicators was estimated. This less restrictive specification relaxed the assumption of equal within-profile variances while retaining the assumption of zero within-profile covariances, thereby allowing greater heterogeneity in the dispersion of the indicators across profiles. As with the primary analysis, information criteria continued to improve as additional profiles were added, including beyond the five-profile solution. Accordingly, the five-profile solution was not selected based on statistical fit indices alone. Rather, consistent with the decision process described above, five profiles were retained based on the joint consideration of parsimony, profile-size adequacy, classification quality, and substantive interpretability. In particular, solutions with more than five profiles introduced increasingly small profiles without providing sufficient substantive differentiation to justify the additional complexity (Table 4).

The robustness of the five-profile classification was further assessed by comparing individual profile assignments across the primary equal-variance specification and the alternative profile-specific variance specification. The Adjusted Rand Index (ARI) was 0.930, indicating a very high degree of correspondence between the two classifications. Overall, 877 of the 917 participants (95.6%) were assigned to the same corresponding profile across the two specifications. The cross-classification matrix showed particularly strong correspondence for Profiles 1, 2, 4, and 5, with most participants retaining their original profile classification. Some reclassification was observed, primarily between Profiles 2 and 3, while a smaller number of participants classified into Profile 5 under one specification were assigned to other profiles under the alternative specification. Overall, these findings provide further evidence that the substantive five-profile classification was highly robust to relaxing the equal-variance assumption, despite the continued improvement in information criteria with increasing numbers of profiles.

**Figure 1:**
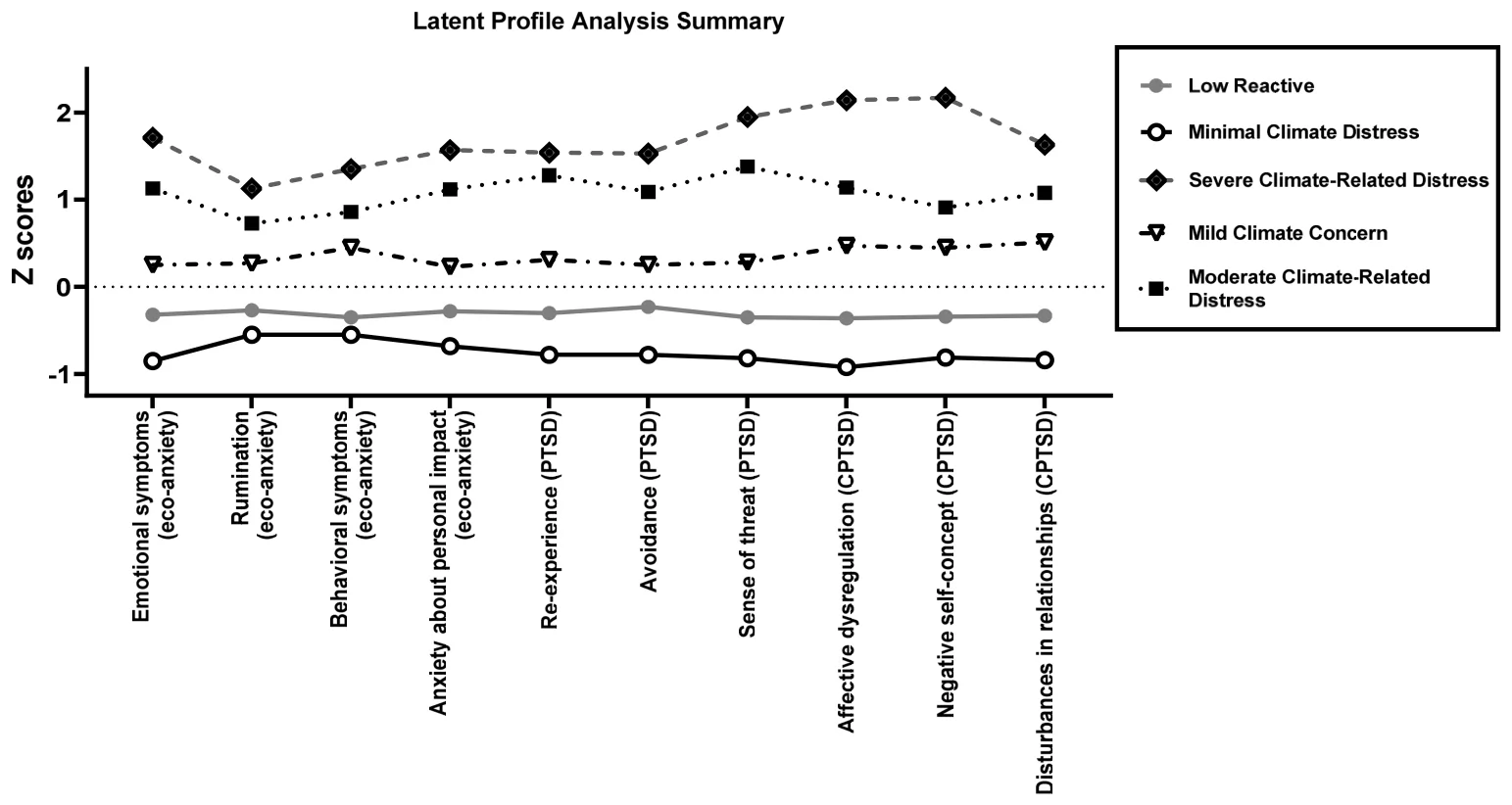
Latent profiles of climate-related distress based on the observed patterns of eco-anxiety, PTSD, and CPTSD symptom dimensions.

As depicted in Figure 1, the five profiles differed in the overall level and configuration of scores across the assessed eco-anxiety dimensions, the three PTSD symptom clusters, and the three CPTSD-related DSO dimensions. Profile 1 (n = 393, 42.9%) represented the largest subgroup and was characterized by scores slightly below the sample mean across most dimensions, with a relatively flat pattern across eco-anxiety and trauma-related indicators. This profile was labeled “Low-Reactive”. Profile 2 (n = 101, 11.0%) showed moderately elevated scores across the assessed domains, with a relatively consistent pattern across eco-anxiety, PTSD, and CPTSD indicators. Compared with Profile 3, scores were generally lower across dimensions. This profile was labeled “Moderate Climate-Related Distress”. Profile 3 (n = 49, 5.3%) was the smallest subgroup and displayed consistently elevated scores across all assessed dimensions. The highest scores were observed for CPTSD-related indicators, followed by PTSD and eco-anxiety measures. This profile was labeled “Severe Climate-Related Distress”. Profile 4 (n = 189, 20.6%) was characterized by scores slightly above the sample mean, particularly on eco-anxiety and CPTSD-related dimensions, whereas PTSD scores remained within the low-to-moderate range. This profile was labeled “Mild Climate Concern”. Finally, Profile 5 (n = 185, 20.2%) exhibited the lowest scores across the assessed indicators, particularly for PTSD and CPTSD, with consistently negative z-scores across these dimensions. Eco-anxiety scores were also comparatively low. This profile was labeled “Minimal Climate Distress”.

### 3.2. Multivariate Analysis of Variance

A MANOVA was performed to assess differences among the five profiles with respect to Connectedness with Nature and Protection of Nature. Preliminary diagnostic analyses indicated that the assumptions of normality, linearity, absence of outliers, homogeneity of variance–covariance matrices, and lack of multicollinearity were adequately satisfied, with no substantive violations detected. Statistically significant differences were identified among the profiles on the combined dependent variables, F(8, 1162) = 4.45, *p* < 0.001, η^2^ = 0.03. Post-hoc univariate analyses with Bonferroni-adjusted significance levels (α = 0.05) indicated no statistically significant differences among profiles in Connectedness with Nature. In contrast, significant differences between profiles were observed for Protection of Nature (Table 5). Scores for Protection of Nature were significantly lower in Profile 5 than in all other profiles (*p* = 0.001–0.009) (Table 6). These findings indicate that Protection of Nature differentiates the profiles, particularly Profile 5, whereas Connectedness with Nature does not show statistically significant between-profile differences. Importantly, these associations should not be interpreted as evidence that either dimension constitutes a resilience or vulnerability factor, as the present cross-sectional analysis does not establish causal or directional relationships.

### 3.3. Multinomial Logistic Regression

Finally, multinomial logistic regression analyses were conducted to examine the associations between latent profile membership, specified as the dependent variable, and a set of sociodemographic, clinical, attachment-related, and childhood adversity variables, specified as predictor variables. Profile membership was defined according to participants’ most likely latent class, based on their posterior class-assignment probabilities. The Minimal Climate Distress profile was specified as the reference category in the multinomial logistic regression model, such that the estimated odds ratios (ORs) represent the odds of membership in each of the remaining profiles relative to this reference profile.

Categorical predictors were entered using binary or dummy coding, as appropriate. For binary sociodemographic and clinical variables, the category representing the characteristic of interest was coded as 1 and the comparison category as 0. Specifically, sex was coded as female = 1 and male = 0; age was dichotomized into young adults aged 18–35 years (coded 1) and non-young adults aged ≥ 36 years (coded 0), consistent with previous research using the same age categorization (e.g., Ferrajão et al., 2026) and with research examining young adults (Lowe et al., 2013). This categorization was adopted to capture the broader developmental period of young adulthood, which encompasses diverse transitional experiences across education, employment, relationships, and family formation. Educational attainment was coded as higher education = 1 and not higher education = 0; professional status as employee = 1 and not employee = 0; and socioeconomic status as medium-high or high = 1 and all other categories = 0. Direct exposure to storms, floods, droughts, and wildfires was coded as yes = 1 and no = 0. Prior psychiatric conditions were similarly coded as yes = 1 and no = 0. Accordingly, ORs greater than 1 indicate higher odds of membership in the respective profile, relative to the reference profile, among participants presenting the characteristic coded as 1.

For the multinomial logistic regression analyses, each attachment style was operationalized as a binary predictor based on its categorical classification (Collins, 1996), such that the variable indicated the presence (1) or absence (0) of the respective attachment style. Accordingly, separate binary indicators were created for secure, preoccupied, dismissing, and fearful attachment, with each predictor representing whether participants met the categorical criteria for that specific attachment style. Thus, the regression coefficients and corresponding ORs reflect the association between the presence of a given attachment style and the odds of the outcome, relative to its absence.

For the multinomial regression analyses, CTQ dimensions were categorized according to established cut-off values for the severity of childhood maltreatment, following D. Bernstein and Fink (1998). For each CTQ domain included in the regression model, categories were recoded into dummy variables, with minimal/low exposure serving as the reference category and moderate/severe exposure as the comparison category. Thus, the corresponding ORs represent the relative odds of membership in each latent profile for participants reporting moderate/severe levels of the respective form of childhood maltreatment compared with those reporting minimal/low levels. This procedure was applied to the relevant CTQ domains, including emotional abuse, physical abuse, and physical neglect. For all predictors, statistical significance was evaluated using a two-sided significance level of α = 0.05. Given the number of predictors and profile-specific contrasts examined, the multinomial regression analyses were considered exploratory. Statistical significance was evaluated at α = 0.05 for individual regression coefficients, and findings were interpreted with caution given the potential for inflated Type I error due to multiple testing.

As shown in Table 7, gender was significantly associated with profile membership, with the magnitude of the associations varying across profiles. Female participants exhibited significantly higher odds of belonging to the Moderate Climate-Related Distress and Mild Climate Concern profiles relative to the reference group, with approximately twofold (OR = 1.97) and more than 1.5-fold higher odds (OR = 1.66), respectively. Age was similarly associated with profile membership. Younger participants had 35% lower odds of membership in the Low-Reactive profile (OR = 0.65), while exhibiting more than threefold higher odds of membership in both the Moderate Climate-Related Distress (OR = 3.16) and Severe Climate-Related Distress (OR = 3.05) profiles. Psychiatric history was also significantly associated with profile membership. A history of psychiatric disorder was associated with lower odds of membership in the Low-Reactive profile (OR = 0.64), corresponding to a 36% reduction in the odds, but with more than two-and-a-half-fold higher odds of membership in the Moderate Climate-Related Distress profile (OR = 2.72).

Regarding direct exposure to extreme weather events, previous experiences of droughts, wildfires, and storms were significantly associated with profile membership. Participants in the Moderate Climate-Related Distress (OR = 1.87) and Mild Climate Concern (OR = 1.81) profiles had nearly twofold higher odds of reporting previous exposure to drought, whereas those in the Low-Reactive profile exhibited lower odds of reporting previous exposure to drought (OR = 0.66), corresponding to a 34% reduction in the odds. Participants in the Severe Climate-Related Distress profile had more than threefold higher odds of reporting previous exposure to storms (OR = 3.30) and nearly twofold higher odds of reporting previous exposure to wildfires OR = 1.89). These findings indicate differential distributions of direct exposure across profiles, rather than establishing exposure to extreme weather events as a determinant of climate-related distress.

Attachment style was also significantly associated with profile membership across secure, preoccupied, and fearful dimensions. Secure attachment was associated with nearly twofold higher odds of membership in the Low-Reactive (OR = 1.83) and Mild Climate Concern (OR = 1.85) profiles. Conversely, secure attachment was associated with markedly lower odds of membership in the Moderate Climate-Related Distress and Severe Climate-Related Distress profiles, with the odds reduced by 79% (OR = 0.21) and 93% (OR = 0.07), respectively. Preoccupied attachment was associated with nearly twofold higher odds of membership in the Moderate Climate-Related Distress profile (OR = 1.95). Fearful attachment was associated with higher odds of membership in the Moderate Climate-Related Distress (OR = 2.68) and Severe Climate-Related Distress (OR = 3.17) profiles, with approximately two-and-a-half-fold and threefold higher odds, respectively. In contrast, fearful attachment was associated with lower odds of membership in the Low-Reactive profile (OR = 0.42), corresponding to a 58% reduction in the odds.

With respect to adverse childhood experiences, emotional abuse, physical neglect, and physical abuse were significantly associated with profile membership. The Low-Reactive profile was associated with lower odds of reporting moderate/severe levels of emotional abuse (OR = 0.42), corresponding to a 58% reduction in the odds relative to the reference category. In contrast, moderate or severe levels of emotional abuse were associated with nearly fourfold higher odds of membership in the Moderate Climate-Related Distress profile (OR = 3.90) and more than sixfold higher odds of membership in the Severe Climate-Related Distress profile (OR = 6.15). Moderate-to-severe physical neglect was associated with more than fivefold higher odds of membership in the Severe Climate-Related Distress profile (OR = 5.65). Moderate-to-severe physical abuse was associated with nearly threefold higher odds of membership in the Mild Climate Concern profile (OR = 2.93).

Given the absence of a universally applicable minimum sample-size criterion for multinomial logistic regression, model adequacy was evaluated based on the total analytic sample, the number of predictors, the distribution of observations across outcome categories and predictor levels, and indicators of parameter-estimation stability. The effective analytic sample was N = 917, corresponding to participants with complete information on the outcome and all covariates included in the model. Model convergence, coefficient estimates, standard errors, and confidence intervals were examined for indications of unstable estimation or sparse-data problems. No substantial estimation problems were identified, and the prespecified predictor set was therefore retained. Nevertheless, the relatively small size of the smallest group profile (n = 49) was considered when interpreting the precision and generalizability of the profile-specific estimates.

To facilitate the interpretation of the multinomial regression findings, Table 8 provides a structured summary of the main predictors of latent profile membership across the identified profiles. Overall, the findings indicate a consistent and graded pattern of associations, with the magnitude and/or direction of several predictors varying systematically across profiles.

## 4. Discussion

Climate change is increasingly recognized as a significant stressor that exacerbates adverse mental health outcomes, which are unevenly distributed across populations (Charlson et al., 2021). As climate-related stressors become more frequent, severe, and prolonged, individuals may experience disruptions to daily life, access to resources, and perceptions of safety, with psychological impacts varying substantially across individuals and contexts (Hayes et al., 2018). Consistent with this background, the present study examined heterogeneity in climate-related distress by identifying distinct profiles based on the co-occurrence of eco-anxiety and ICD-11 PTSD and CPTSD-related DSO symptom clusters.

Five distinct profiles emerged, ranging from minimal symptom endorsement to configurations characterized by elevated levels of eco-anxiety, PTSD symptom clusters, and DSO symptoms. Thus, the hypothesized four- to five-profile solution was supported. The hypothesis that environmental identity dimensions would differ across profiles received partial support: Protection of Nature differed significantly across profiles, whereas Connectedness with Nature did not. Similarly, the hypothesis that vulnerability- and resilience-related characteristics would differ across profiles received partial support. Significant differences in profile membership were observed as a function of age, gender, direct exposure to climate-related events, prior psychopathology, attachment, and childhood adversity, whereas socioeconomic status, educational level, employment status, and living environment were not significantly associated with profile membership.

To the best of our knowledge, this is the first study to use latent LPA to examine the co-occurring patterns of eco-anxiety, PTSD symptom clusters, and DSO symptoms in relation to CC. By considering these domains simultaneously, the findings extend previous person-centred research and suggest that climate-related distress may be expressed through qualitatively distinct symptom configurations. Taken together, these findings support a biopsychosociocultural and trauma-informed conceptualization of climate-related distress. Differences in profile membership were associated not only with exposure to climate-related events, but also with developmental, psychological, demographic, and environmental factors. As indicated by O’Donnell and Palinkas (2024) and Shaligram et al. (2025), this pattern suggests that vulnerability and resilience to climate-related distress reflect the interplay of multiple and potentially cumulative correlates.

### 4.1. Profiles of PTSD, DSO and Eco-Anxiety Symptoms

Five distinct profiles emerged from the LPA, differing in both the overall severity and configuration of eco-anxiety, PTSD symptom clusters, and DSO symptoms. Profile 5 (Minimal Climate Distress) and Profile 3 (Severe Climate-Related Distress) represented the contrasting ends of the observed symptom distribution, with the lowest and highest levels of symptom endorsement, respectively. The five-profile solution was consistent with the study hypothesis and with previous person-centered research (e.g., Çimen et al., 2025; Ferrajão et al., 2026; Zhen et al., 2020) demonstrating substantial heterogeneity in eco-anxiety and trauma-related responses. Importantly, these profiles should be interpreted as empirically derived patterns of symptom endorsement rather than as distinct clinical or diagnostic categories.

Low-distress profiles, namely Profile 5 (Minimal Climate Distress) and Profile 1 (Low-Reactive), were characterized by comparatively low levels of eco-anxiety, PTSD symptoms, and DSO symptoms. Based on previous research, one possible interpretation is that low symptom activation may reflect lower perceived threat, concern, or psychological engagement with climate change (CC) (Shomuyiwa & Lucero-Prisno, 2025; Stanley et al., 2021). However, disengagement, denial, or dissociation were not directly assessed and therefore cannot be inferred from profile membership. This distinction is important because previous research has shown that moderate levels of eco-anxiety may coexist with preserved self-efficacy, adaptive coping, and pro-environmental engagement (Ferrajão et al., 2026). Accordingly, very low symptom activation should not necessarily be interpreted as indicative of optimal adaptation.

Profile 4 (Mild Climate Concern) was characterized by elevated levels of eco-anxiety and DSO symptoms, alongside comparatively lower levels of core PTSD symptom clusters. This configuration may reflect heightened sensitivity to climate-related threats without the broader symptom burden observed in the higher-distress profiles. The co-occurrence of eco-anxiety and DSO symptoms is consistent with previous work highlighting moral, relational, and identity-related dimensions of climate distress, including feelings of responsibility, guilt, powerlessness, and frustration in response to environmental degradation and societal inaction (Banwell & Eggert, 2024). Although these processes were not directly assessed in the present study, they provide plausible hypotheses for future research investigating the mechanisms underlying the co-occurrence of eco-anxiety and DSO symptoms.

Profiles 2 (Moderate Climate-Related Distress) and 3 (Severe Climate-Related Distress) showed the highest overall symptom burden. Profile 2 was characterized by consistently elevated symptoms across domains, with relatively pronounced core PTSD symptoms, particularly re-experiencing and a heightened sense of current threat. This pattern is consistent with previous person-centered studies identifying profiles in which core PTSD symptoms are more prominent than DSO symptoms (Çimen et al., 2025; Teysseyre et al., 2026). It is also consistent with evidence that the perception of an ongoing threat is associated with greater persistence of post-traumatic stress responses (Johnson et al., 2024). Profile 2 may therefore represent a configuration in which climate-related distress is particularly characterized by threat-related symptom activation, although the cross-sectional design does not permit conclusions regarding symptom development or maintenance over time.

Lastly, Profile 3 was distinguished by markedly elevated DSO symptoms, together with high levels of core PTSD symptom clusters and eco-anxiety. The prominence of DSO symptoms differentiates this configuration from profiles characterized primarily by elevated core PTSD symptoms and is consistent with previous person-centered research identifying symptom profiles marked by more pronounced complex-trauma dimensions (Çimen et al., 2025; Teysseyre et al., 2026). The co-occurrence of elevated eco-anxiety, core PTSD symptoms, and DSO symptoms may be particularly relevant in light of previous work suggesting that higher levels of eco-anxiety are associated with heightened perceptions of threat and uncertainty, as well as impaired psychological functioning (Ferrajão et al., 2026). Moreover, climate-related stressors may interact with concurrent or pre-existing vulnerabilities, potentially contributing to greater difficulties with emotion regulation, self-perception, and interpersonal relationships among individuals in this profile (O’Donnell & Palinkas, 2024; Woodbury, 2019; Shomuyiwa & Lucero-Prisno, 2025).

Although Profile 3 should not be interpreted as a diagnostic category, the marked elevation of DSO symptoms within this high-distress configuration highlights the potential relevance of complex-trauma symptom dimensions for understanding severe forms of climate-related distress. This finding warrants particular attention given that CPTSD symptomatology has been associated with greater functional impairment and psychiatric comorbidity than PTSD alone (Brewin, 2020; World Health Organization, 2022).

### 4.2. Vulnerability and Resilience Characteristics

The hypothesis that vulnerability- and resilience-related characteristics would differ across profiles was partially supported. Higher-distress profiles were characterized by greater exposure to climate-related events, childhood adversity, insecure attachment, and prior psychopathology, whereas lower-distress profiles were more strongly associated with secure attachment and no history of prior mental health difficulties. Taken together, these associations are consistent with the trauma and adversity framework proposed by O’Donnell and Palinkas (2024), which posits that responses to climate-related stressors may be shaped by the interplay between pre-existing vulnerabilities and psychological and social resources.

Direct exposure to extreme climate events was significantly associated with profile membership and was more prevalent among the higher-distress profiles. This finding is consistent with previous studies linking direct exposure to extreme weather events with greater psychological distress and PTSD symptoms (Barbani, 2025; Chique et al., 2021). In the present sample, the events associated with higher-distress profiles included climate-related events that have caused substantial disruption in Portugal, including disturbances to daily life, property damage, economic losses, and loss of life (Carvalho et al., 2014). These findings are therefore consistent with previous evidence indicating that the psychological burden associated with environmental disasters may be greater among individuals experiencing more direct or consequential exposure.

Developmental adversity was also strongly associated with the higher-distress profiles. Developmental adversity was also strongly associated with the higher-distress profiles. Moderate-to-severe emotional abuse and physical neglect were particularly associated with membership in the Moderate and Severe Climate-Related Distress profiles. These findings are consistent with longitudinal evidence showing cumulative, dose-related associations between childhood trauma and poorer mental health responses to later disaster exposure (Liang et al., 2023), as well as with broader trauma research linking cumulative adversity with PTSD and CPTSD symptomatology (Karatzias et al., 2019; Hyland et al., 2017; Li et al., 2023). The prominence of DSO symptoms in the Severe Climate-Related Distress profile is especially noteworthy in this context, given the established association between DSO symptoms and prolonged, repeated, and developmental adversity. Moderate-to-severe physical abuse was also associated with the Mild Climate Concern profile, suggesting that childhood adversity was not restricted to profiles with the highest overall symptom burden.

Attachment further differentiated the profiles. Secure attachment was associated with the lower-distress profiles and with Mild Climate Concern, whereas fearful attachment was associated with the Severe Climate-Related Distress profile and preoccupied attachment with the Moderate Climate-Related Distress profile. These findings are consistent with previous evidence linking secure attachment with more adaptive emotion regulation and support-seeking, and insecure attachment with greater post-traumatic symptom burden following adversity (Morison & Benight, 2022; Woodhouse et al., 2015). Considered alongside the associations with childhood adversity and direct exposure to climate-related events, these findings are compatible with the view that responses to current climate-related stressors are embedded within prior developmental histories. Theoretical accounts have proposed that climate-related threats may reactivate or amplify pre-existing trauma-related vulnerabilities (Woodbury, 2019).

A history of psychiatric disorder was associated with higher odds of membership in the Moderate Climate-Related Distress profile, whereas the absence of previous psychiatric difficulties was associated with the Low-Reactive profile. This finding is consistent with previous prospective and disaster-related research indicating that pre-existing mental health difficulties are associated with poorer psychological outcomes following extreme events (Tang et al., 2017; Yakubu et al., 2026). Evidence suggests that individuals with a history of mental health difficulties may experience greater challenges in adapting to additional stressors and to the uncertainty associated with climate change (CC) threats, potentially increasing vulnerability to psychological distress and trauma-related symptomatology. Moreover, pre-existing mental health conditions are often associated with alterations in stress responses, which may further affect adaptation to climate-related challenges (Tang et al., 2017; Yakubu et al., 2026).

Age and gender also differentiated profile membership. Younger age was more strongly associated with the higher-distress profiles, consistent with previous evidence indicating greater climate-related concern and psychological distress among younger populations (Clayton et al., 2023). As those likely to experience the cumulative and long-term consequences of a changing climate, younger generations may face sustained uncertainty regarding future environmental, social, and economic conditions. Furthermore, greater awareness of current and anticipated climate-related threats may contribute to heightened levels of psychological distress and trauma-related symptomatology (Clayton et al., 2023). In turn, female sex was associated with higher odds of membership in the Moderate Climate-Related Distress and Mild Climate Concern profiles relative to male sex. This pattern is consistent with previous research indicating greater climate-related distress among women and should be considered within the context of pre-existing social and structural inequalities, including unequal access to resources, decision-making processes, and economic opportunities (Adams et al., 2021). Conversely, previous studies have reported lower levels of climate concern and emotional engagement with climate issues among men, as well as greater endorsement of CC denial and greater confidence in institutional responses (Clayton et al., 2023). These patterns may reduce the perceived relevance of environmental threats and, consequently, levels of distress, although such mechanisms were not directly assessed in the present study and therefore cannot be inferred from the current findings.

In contrast, socioeconomic status, educational level, employment status, and living environment were not significantly associated with profile membership. These findings differ from previous studies identifying socioeconomic disadvantage as a correlate of poorer mental health outcomes following climate-related disasters (Gauthier et al., 2025; Yakubu et al., 2026). The relatively homogeneous socioeconomic composition of the present sample may have reduced variability in these characteristics and, consequently, limited the ability to detect meaningful differences in profile membership.

Furthermore, the hypothesis concerning environmental identity was partially supported. Our results did not reveal statistically significant differences across profiles in the Connectedness with Nature dimension. However, the Minimal Climate Distress profile exhibited significantly lower scores on Nature Protection than the other profiles, which is consistent with the interpretation of this profile as reflecting lower psychological engagement with CC-related issues. Previous research has consistently shown that lower levels of connectedness with nature are associated with reduced concern about environmental degradation and lower engagement in pro-environmental behaviors (Barrable & Booth, 2022; Martin et al., 2020). Conversely, stronger connectedness with nature and environmental engagement may represent ways of coping with climate-related distress by supporting meaning-making and a sense of agency (Herman, 2015).

However, the present findings suggest that connectedness with nature does not necessarily correspond to lower psychological distress. Rather, among individuals experiencing severe climate-related trauma symptomatology, stronger environmental identification may coexist with both adaptive and maladaptive psychological responses, particularly when heightened awareness of environmental degradation and ecological loss contributes to greater emotional distress (Clayton & Karazsia, 2020; Pihkala, 2020). The significant differences observed in Protection of Nature further support this interpretation. Whereas individuals in the Minimal Climate Distress profile reported the lowest levels of Protection of Nature, those in the Severe Climate-Related Distress profile exhibited the highest levels. Although causal conclusions cannot be drawn, these findings suggest that greater climate-related distress may coexist with heightened environmental concern and engagement, potentially reflecting efforts to maintain or restore a sense of agency in response to climate-related threats (Herman, 2015). Such engagement may nevertheless coexist with elevated psychological distress, particularly when environmental degradation is perceived as an ongoing and uncontrollable threat (Clayton & Karazsia, 2020; Thomson & Roach, 2023).

Taken together, these findings provide important clarification regarding the role of CC in severe climate-related distress. Rather than supporting a simple distinction between CC as a primary traumatizing event and a secondary catalyst, our findings are more consistent with an interactional and cumulative model. Climate-related stressors may constitute potentially traumatic experiences in their own right, while their psychological impact may also be shaped by pre-existing developmental and psychological vulnerabilities (O’Donnell & Palinkas, 2024). The prominent association between emotional abuse and physical neglect and membership in the Severe Climate-Related Distress profile suggests that earlier adversity may constitute an important vulnerability context in which subsequent climate-related threats are experienced and processed. This interpretation is consistent with evidence linking childhood adversity to poorer psychological responses to subsequent disaster exposure (Liang et al., 2023). Importantly, however, the cross-sectional design does not allow us to determine whether climate-related stressors reactivate pre-existing trauma-related processes or whether developmental adversity causally increases susceptibility to climate-related distress. Thus, the findings should be interpreted as evidence of an association between cumulative vulnerability and severe climate-related distress, rather than as evidence of a specific temporal or causal pathway.

### 4.3. Limitations

The present findings should be interpreted in light of several limitations. First, the cross-sectional design precludes causal inference and limits conclusions regarding the temporal relationships between vulnerability, resilience, and climate-related psychological outcomes. Longitudinal studies are therefore needed to examine the stability of the identified profiles and clarify the temporal dynamics of climate-related distress. Second, the use of convenience sampling through online platforms and social media may have introduced self-selection and coverage biases, limiting generalizability. The sample was predominantly highly educated and may therefore underrepresent older adults and rural populations. Accordingly, the findings should not be considered representative of the Portuguese population. The non-significant association involving rural residence should also be interpreted cautiously given the restricted representation of rural participants. Third, reliance exclusively on self-report measures may have increased susceptibility to response biases and common method variance, particularly because predictors and outcomes were assessed using the same method. Future research should incorporate multi-method approaches and, where feasible, objective or behavioral indicators. A further limitation concerns the treatment of latent profile membership in the analyses involving external variables. Participants were assigned to their most likely profile based on posterior probabilities, which does not fully account for individual-level classification uncertainty. Although the profile solution showed good classification quality and robustness in additional analyses, some degree of uncertainty in profile assignment remains. Consequently, associations between profile membership and external variables should be interpreted with this consideration in mind. Future research could address this issue using three-step or BCH-type approaches that explicitly account for classification uncertainty. The multinomial regression findings should be interpreted cautiously given the number of predictors and profile-specific contrasts examined. The absence of an adjustment for multiple comparisons represents an additional limitation, as it increases the possibility of an inflated Type I error rate. Accordingly, these findings should be considered exploratory, particularly those associated with *p*-values close to 0.05. Finally, the latent profiles and their predictors were examined within the same sample. Although the identified profiles provide an informative characterization of heterogeneity, their stability and external validity require replication in independent and more representative samples. Future research should therefore seek to reproduce the profile structure across populations and settings and further examine the processes underlying climate-related distress and eco-anxiety.

## 5. Conclusions

This study provides preliminary evidence of heterogeneity in climate-related distress, identifying empirically derived profiles characterized by distinct configurations and levels of co-occurring eco-anxiety and trauma-related symptoms, ranging from relatively low symptom endorsement to more pronounced symptom elevations across symptom domains. Furthermore, profile membership was associated with several individual and contextual factors. Female gender, younger age, cumulative trauma exposure, prior psychopathology, and insecure attachment were associated with membership in higher-distress profiles, whereas male gender, older age, the absence of prior mental health difficulties, and secure attachment were associated with membership in lower-distress profiles.

These results extend current knowledge of psychological responses to CC, support the relevance of person-centered approaches, and identify potential correlates of distinct symptom configurations. Collectively, these findings may have important implications for climate adaptation and mental health responses. In particular, attachment emerged as a relevant correlate of profile membership and warrants further examination of its potential role in psychological adjustment. Protection of Nature, but not Connectedness with Nature, significantly differentiated the identified profiles, with the Minimal Climate Distress profile showing lower levels of Protection of Nature than all other profiles. Further research should examine the stability and development of these profiles over time and investigate whether attachment- and nature-related processes may inform evidence-based prevention and intervention strategies.

## Figures and Tables

**Table 1:** Sample demographic characteristics.

Variables	*n*	%
**Sex**		
Female	579	63.1
Male	338	36.9
**Civil status**		
Single	507	55.3
Married or cohabitation	321	35.0
Divorced	77	8.4
Widowed	12	1.3
**Education**		
Less than nine years of education	17	1.9
Nine years of education	36	3.9
12 years of education	449	49.0
Higher education	415	45.2
**Professional status**		
Student	381	41.6
Employee	438	47.8
Unemployed	62	6.8
Retired	36	3.9
**Socioeconomic status**		
Low	76	8.3
Medium	643	70.1
Medium–high	182	19.8
High	16	1.7

**Table 2:** Factor loadings for the adapted version of the International Trauma Questionnaire.

**PTSD Symptoms**					
**Re-Experiencing**		**Avoidance**		**Sense of Threat**	
**Item**	**Standardized Factor Loadings/(SE)**	**Item**	**Standardized Factor Loadings/(SE)**	**Item**	**Standardized Factor Loadings/(SE)**
1	0.37/(0.02) ***	4	0.46/(0.03) ***	7	0.79/(0.04) ***
2	0.47/(0.03) ***	5	0.79/(0.04) ***	8	0.83/(0.04) ***
3	0.53/(0.04) ***	6	0.91/(0.04) ***	9	0.74/(0.04) ***
**CPTSD Symptoms**					
**Affect Dysregulation**		**Negative Self-Concept**		**Disturbances in Relationships**	
**Item**	**Standardized Factor Loadings/(SE)**	**Item**	**Standardized Factor Loadings/(SE)**	**Item**	**Standardized Factor Loadings/(SE)**
10	0.68/(0.04) ***	13	0.67/(0.03) ***	16	0.86/(0.04) ***
11	0.63/(0.04) ***	14	0.64/(0.03) ***	17	0.73/(0.03) ***
12	0.58/(0.03) ***	15	0.92/(0.03) ***	18	0.60/(0.03) ***

Note. SE = Standard error; *** *p* < 0.001.

**Table 3:** Fit indices for the latent profile analysis.

Model	AIC	BIC	SSABIC	BLRT	*p*	Entropy	Smallest Group Proportion
2 classes	22,509.87	22,659.29	22,560.84	3537.09	0.001	0.94	24.8
3 classes	21,520.41	21,722.85	21,589.46	1011.46	0.001	0.88	13.0
4 classes	20,984.13	21,239.59	21,071.27	558.28	0.001	0.88	7.3
5 classes	20,728.76	21,037.24	20,833.98	277.37	0.001	0.85	5.2
6 classes	20,644.95	21,006.45	20,768.26	105.81	0.001	0.83	3.1
7 classes	20,267.91	20,682.43	20,409.31	399.04	0.001	0.85	1.7
8 classes	20,069.38	20,536.92	20,228.86	220.53	0.001	0.87	1.7

Note. AIC = Akaike information criterion; BIC = Bayesian information criterion; SSABIC = Sample-size-adjusted Bayesian information criterion; and BLRT = Bootstrap likelihood ratio test.

**Table 4:** Fit indices for the latent profile analysis.

Model	AIC	BIC	SSABIC	BLRT	*p*	Entropy	Smallest Group Proportion
2 classes	21,378.25	21,575.92	21,445.71	4720.32	0.001	0.94	33.1
3 classes	19,532.93	19,831.84	19,634.94	1884.07	0.001	0.92	16.4
4 classes	17,018.07	17,418.22	17,154.62	2556.86	0.001	0.96	10.0
5 classes	16,837.52	17,088.21	16,923.07	812.41	0.001	0.92	5.7
6 classes	16,711.88	17,005.97	16,812.24	443.63	0.001	0.84	3.2
7 classes	14,736.11	15,035.02	14,838.12	184.32	0.001	0.82	1.6
8 classes	14,626.40	14,963.88	14,741.57	125.71	0.001	0.82	1.6

Note. AIC = Akaike information criterion; BIC = Bayesian information criterion; SSABIC = sample-size-adjusted Bayesian information criterion; and BLRT = Bootstrap likelihood ratio test.

**Table 5:** Tests of between-subject effects.

Dependent Variables	Mean Square	df	F	*p*	Partial Eta Squared	Observed Power
Connectedness with nature	303.46	4	1.64	0.16	0.01	0.51
Protection of nature	221.95	4	6.76	0.001	0.05	0.99

**Table 6:** Means and standard deviations of profiles on environmental identity dimensions.

Profile	Connectedness with Nature			Protection of Nature		
	M	SD	95% CI	M	SD	95% CI
Profile 1: Low-Reactive	53.23	14.14	51.68; 54.98	16.95	5.76	16.28; 17.62
Profile 2: Moderate Climate-Related Distress	55.57	13.17	52.16; 58.97	18.26	5.06	16.97; 19.56
Profile 3: Severe Climate-Related Distress	57.46	13.19	52.13; 62.79	20.19	6.22	17.68; 22.70
Profile 4: Mild Climate Concern	55.76	12.90	53.49; 58.02	17.54	6.29	16.45; 18.63
Profile 5: Minimal Climate Distress	52.34	13.09	49.57; 55.12	14.53	5.20	13.43; 15.62

**Table 7:** Results of the multinomial logistic regression predicting latent profile membership.

**Profile 1: Low-Reactive**					
**Effect**	**−2 Log Likelihood**	**Chi-Square**	**df**	***p***	**OR (95% CI)**
Intercept	567.39	0.00	0		
Gender (female)	567.83	0.13	1	0.72	1.07 (0.75; 1.52)
Age (young)	572.56	4.09	1	0.04	0.65 (0.44; 0.98)
Education (low)	566.78	0.60	1	0.44	0.74 (0.34; 1.59)
Professional status (not employed)	570.54	3.05	1	0.08	0.70 (0.47; 1.04)
Socioeconomic status (low)	567.64	0.06	1	0.81	0.94 (0.59; 1.52)
Living environments (rural)	570.77	3.31	1	0.07	1.37 (0.98; 1.93)
Prior psychiatric conditions (yes)	571.95	4.73	1	0.03	0.64 (0.43; 0.96)
Flood (yes)	567.31	0.02	1	0.89	1.03 (0.70; 1.52)
Droughts (yes)	572.81	5.36	1	0.02	0.66 (0.46; 0.94)
Wildfire (yes)	567.33	0.19	1	0.66	0.91 (0.59; 1.40)
Storms (yes)	567.26	0.02	1	0.88	0.96 (0.54; 1.68)
Extreme temperatures (yes)	567.99	0.65		0.42	2.50 (0.27; 11.20)
Secure (yes)	575.56	13.25	1	0.001	1.83 (1.32; 2.54)
Preoccupied (yes)	567.44	0.01	1	0.91	1.02 (0.66; 1.58)
Dismissing (yes)	569.04	1.12	1	0.29	1.30 (0.80; 2.10)
Fearful (yes)	573.42	5.97	1	0.01	0.42 (0.27; 0.65)
Emotional abuse (moderate/severe)	572.99	5.51	1	0.02	0.42 (0.20; 0.87)
Physical abuse (moderate/severe)	568.19	0.45	1	0.50	0.58 (0.22; 2.22)
Sexual abuse (moderate/severe)	567.29	0.01	1	0.92	0.95 (0.38; 2.41)
Emotional neglect (moderate/severe)	569.07	1.96	1	0.16	0.61 (0.31; 1.22)
Physical neglect (moderate/severe)	567.84	0.27	1	0.60	0.78 (0.31; 1.99)
**Profile 2: Moderate Climate-Related Distress**					
**Effect**	**−2 Log Likelihood**	**Chi-Square**	**df**	***p***	**OR (95% CI)**
Intercept	235.96	0.00	0		
Gender (female)	241.58	4.28	1	0.04	1.97 (1.04; 3.73)
Age (young)	243.16	10.45	1	0.001	3.16 (1.92; 5.21)
Education (low)	237.06	0.95	1	0.33	1.48 (0.67; 3.22)
Professional status (not employed)	237.26	1.47	1	0.23	1.45 (0.80; 2.63)
Socioeconomic status (low)	237.68	1.85	1	0.17	1.59 (0.82; 3.09)
Living environments (rural)	236.62	0.39	1	0.54	1.20 (0.68; 2.13)
Prior psychiatric conditions (yes)	243.12	9.00	1	0.001	2.72 (1.42; 5.23)
Flood (yes)	235.61	0.16	1	0.69	1.14 (0.61; 2.12)
Droughts (yes)	241.39	4.29	1	0.04	1.87 (1.04; 2.95)
Wildfire (yes)	235.92	0.08	1	0.78	1.09 (0.59; 2.03)
Storms (yes)	236.14	0.22	1	0.64	1.16 (0.51; 2.63)
Extreme temperatures (yes)	236.83	0.80	1	0.37	1.77 (0.30; 5.74)
Secure (yes)	243.26	18.59	1	0.001	0.21 (0.10; 0.43)
Preoccupied (yes)	242.05	4.48	1	0.03	1.95 (1.05; 3.61)
Dismissing (yes)	236.97	0.18	1	0.67	1.18 (0.56; 2.50)
Fearful (yes)	243.05	11.23	1	0.001	2.68 (1.51; 4.77)
Emotional abuse (moderate/severe)	243.43	10.34	1	0.001	3.90 (1.70; 8.92)
Physical abuse (moderate/severe)	235.88	0.10	1	0.75	1.18 (0.41; 3.39)
Sexual abuse (moderate/severe)	236.91	0.88	1	0.35	1.72 (0.55; 5.37)
Emotional neglect (moderate/severe)	235.47	0.01	1	0.94	1.04 (0.43; 2.52)
Physical neglect (moderate/severe)	237.47	1.65	1	0.20	2.06 (0.68; 6.19)
**Profile 3: Severe Climate-Related Distress**					
**Effect**	**−2 Log Likelihood**	**Chi-Square**	**df**	***p***	**OR (95% CI)**
Intercept	97.85	0.00	0		
Gender (female)	99.24	1.51	1	0.22	1.49 (0.79; 2.81)
Age (young)	102.16	9.55	1	0.001	3.05 (1.50; 6.19)
Education (low)	97.46	0.01	1	0.92	1.07 (0.32; 3.55)
Professional status (not employed)	98.54	0.51	1	0.48	1.23 (0.49; 2.19)
Socioeconomic status (low)	99.29	1.60	1	0.21	1.69 (0.75; 3.83)
Living environments (rural)	97.99	0.11	1	0.74	1.14 (0.51; 2.57)
Prior psychiatric conditions (yes)	99.45	2.25	1	0.13	2.11 (0.80; 5.60)
Flood (yes)	98.37	0.27	1	0.60	1.27 (0.52; 2.07)
Droughts (yes)	98.31	0.20	1	0.65	1.20 (0.55; 2.64)
Wildfire (yes)	99.97	4.22	1	0.04	1.89 (1.08; 3.53)
Storms (yes)	102.20	4.34	1	0.001	3.30 (1.34; 9.76)
Extreme temperatures (yes)	98.41	0.45	1	0.52	1.42 (0.57; 2.27)
Secure (yes)	104.28	14.06	1	0.001	0.07 (0.02; 0.28)
Preoccupied (yes)	98.49	0.49	1	0.49	1.55 (0.46; 5.25)
Dismissing (yes)	98.03	0.14	1	0.71	1.23 (0.41; 3.66)
Fearful (yes)	100.02	4.06	1	0.04	3.17 (1.07; 9.90)
Emotional abuse (moderate/severe)	102.85	9.02	1	0.001	6.15 (3.31; 11.41)
Physical abuse (moderate/severe)	99.06	1.13	1	0.29	2.33 (0.49; 11.15)
Sexual abuse (moderate/severe)	98.72	0.68	1	0.41	1.92 (0.41; 9.03)
Emotional neglect (moderate/severe)	97.95	0.10	1	0.75	1.21 (0.37; 3.98)
Physical neglect (moderate/severe)	102.62	7.24	1	0.001	5.65 (1.60; 9.94)
**Profile 4: Mild Climate Concern**					
**Effect**	**−2 Log Likelihood**	**Chi-Square**	**df**	***p***	**OR (95% CI)**
Intercept	431.17	0.00	0		
Gender (female)	436.41	5.10	1	0.02	1.66 (1.07; 2.59)
Age (young)	434.51	3.33	1	0.07	1.53 (0.97; 2.42)
Education (low)	434.03	2.86	1	0.09	1.87 (0.91; 3.85)
Professional status (not employed)	431.92	1.06	1	0.30	1.26 (0.81; 1.97)
Socioeconomic status (low)	431.64	0.55	1	0.46	1.24 (0.70; 2.18)
Living environments (rural)	432.74	2.09	1	0.15	1.36 (0.90; 2.07)
Prior psychiatric conditions (yes)	431.06	0.02	1	0.90	1.04 (0.58; 1.87)
Flood (yes)	431.86	1.03	1	0.31	1.27 (0.80; 2.01)
Droughts (yes)	437.63	7.18	1	0.01	1.81 (1.17; 2.80)
Wildfire (yes)	432.55	1.70	1	0.20	1.41 (0.84; 2.38)
Storms (yes)	432.37	1.38	1	0.24	1.46 (0.78; 2.77)
Extreme temperatures (no)	432.36	1.37	1	0.24	1.44 (0.73; 2.71)
Secure (yes)	435.68	4.39	1	0.04	1.85 (1.04; 3.28)
Preoccupied (yes)	431.88	0.01	1	0.93	1.03 (0.55; 1.93)
Dismissing (yes)	433.28	2.36	1	0.12	1.53 (0.89; 2.61)
Fearful (no)	431.43	0.29	1	0.59	1.20 (0.62; 2.32)
Emotional abuse (moderate/severe)	434.01	2.89	1	0.09	1.84 (0.91; 3.72)
Physical abuse (moderate/severe)	435.89	4.27	1	0.04	2.93 (1.06; 8.14)
Sexual abuse (moderate/severe)	431.28	0.05	1	0.83	1.10 (0.44; 2.77)
Emotional neglect (moderate/severe)	430.51	0.01	1	0.99	1.01 (0.50; 2.01)
Physical neglect (moderate/severe)	433.64	2.63	1	0.11	0.42 (0.15; 1.20)

Note. Reference group = Minimal climate distress profile; *n* = 917. Nagelkerke R^2^ = 0.29.

**Table 8:** Summary of the key predictors of latent profile membership and the observed dose–response pattern across profiles.

LPA Profile and Size	Sociodemographic and Clinical Predictors	Environmental Exposure and Past Trauma Predictors	Conceptual Meaning
Profile 1: Low-reactive (42.9%)	Younger age (OR = 0.65) Prior psychiatric history (OR = 0.64) Secure attachment (OR = 1.83)	Direct exposure to droughts (OR = 0.66) History of emotional abuse (OR = 0.42)	Low emotional/traumatic reactivity; distance from climate threat
Profile 4: Mild climate concern (20.6%)	Female gender (OR = 1.66) Secure attachment (OR = 1.85)	Direct exposure to droughts (OR = 1.81) History of childhood physical abuse (OR = 2.93)	Subclinical, “moral” eco-anxiety; high awareness without functional impairment
Profile 2: Moderate climate-related distress (11.0%)	Younger age (OR = 3.16) Female gender (OR = 1.97) Prior psychiatric history (OR = 2.72) Insecure attachment: Secure (OR = 0.21), Preoccupied (OR = 1.95) and Fearful (OR = 2.68)	Direct exposure to droughts (OR = 1.87) History of childhood emotional abuse (OR = 3.90)	Clinically relevant distress marked by core PTSD threat and re-experiencing
Profile 3: Severe climate-related distress (5.3%)	Younger age (OR = 3.05) Insercure attachment: Secure (OR = 0.07), Fearful (OR = 3.17)	Direct exposure to wildfires (OR = 1.89) and storms (OR = 3.30) Severe childhood emotional abuse (OR = 6.15) and physical neglect (OR = 5.65)	True complex climate trauma; severe emotional dysregulation (DSO) triggered by acute hazards on fragile baselines

## Data Availability

The data presented in this study are available upon request from the corresponding author due to data protection regulations.

## References

[B1-behavsci-16-01801] Adams H., Blackburn S., Mantovani N. (2021). Psychological resilience for climate change transformation: Relational, differentiated and situated perspectives. Current Opinion in Environmental Sustainability.

[B2-behavsci-16-01801] Albrecht G. (2019). Earth emotions: New words for a new world.

[B3-behavsci-16-01801] Ágoston C., Balázs B., Mónus F., Varga A. (2024). Age differences and profiles in pro-environmental behavior and eco-emotions. International Journal of Behavioral Development.

[B4-behavsci-16-01801] Banwell N., Eggert N. (2024). Rethinking ecoanxiety through environmental moral distress: An ethics reflection. The Journal of Climate Change and Health.

[B5-behavsci-16-01801] Barbani S. M. (2025). The indirect consequences of climate change and extreme environmental events on mental health: A literature review. Journal of Public Health.

[B6-behavsci-16-01801] Barrable A., Booth D. (2022). Disconnected: What can we learn from individuals with very low nature connection?. International Journal of Environmental Research and Public Health.

[B7-behavsci-16-01801] Bernstein D., Fink L. (1998). Childhood trauma questionnaire: A retrospective self-report.

[B8-behavsci-16-01801] Bernstein D. P., Stein J. A., Newcomb M. D., Walker E., Pogge D., Ahluvalia T., Stokes J., Handelsman L., Medrano M., Desmond D., Zule W. (2003). Development and validation of a brief screening version of the childhood trauma questionnaire. Child Abuse & Neglect.

[B9-behavsci-16-01801] Bertin F., Clarys D., Tapia G., Chami L., El-Hage W., Vancappel A. (2025). Are all PTSD cases complex PTSD? Results from a latent profile analysis. European Journal of Trauma & Dissociation.

[B10-behavsci-16-01801] Betro S. (2024). From eco-anxiety to eco-hope: Surviving the climate change threat. Frontiers in Psychiatry.

[B11-behavsci-16-01801] Brewin C. R. (2020). Complex post-traumatic stress disorder: A new diagnosis in ICD-11. BJPsych Advances.

[B12-behavsci-16-01801] Campbell-Lendrum D., Neville T., Schweizer C., Neira M. (2023). Climate change and health: Three grand challenges. Nature Medicine.

[B13-behavsci-16-01801] Canavarro M. C. (1997). Relações afetivas e saúde mental: O papel do apego *[Affective relationships and mental health: The role of attachment]*. Ph.D. thesis.

[B14-behavsci-16-01801] Carvalho A., Schmidt L., Santos F. D., Delicado A. (2014). Climate change research and policy in Portugal. Wiley Interdisciplinary Reviews. Climate Change.

[B15-behavsci-16-01801] Charlson F., Ali S., Benmarhnia T., Pearl M., Massazza A., Augustinavicius J., Scott J. G. (2021). Climate change and mental health: A scoping review. International Journal of Environmental Research and Public Health.

[B16-behavsci-16-01801] Chique C., Hynds P., Nyhan M. M., Lambert S., Boudou M., O’Dwyer J. (2021). Psychological impairment and extreme weather event (EWE) exposure, 1980–2020: A global pooled analysis integrating mental health and well-being metrics. International Journal of Hygiene and Environmental Health.

[B17-behavsci-16-01801] Cianconi P., Betrò S., Janiri L. (2020). The impact of climate change on mental health: A systematic descriptive review. Frontiers in Psychiatry.

[B18-behavsci-16-01801] Cianconi P., Hanife B., Grillo F., Betro’ S., Lesmana C. B. J., Janiri L. (2023). Eco-emotions and psychoterratic syndromes: Reshaping mental health assessment under climate change. The Yale Journal of Biology and Medicine.

[B19-behavsci-16-01801] Ciullo A., Franzke C. L. E., Scheffran J., Sillmann J. (2025). Climate-driven systemic risk to the sustainable development goals. PLoS Climate.

[B20-behavsci-16-01801] Clayton S. (2020). Climate anxiety: Psychological responses to climate change. Journal of Anxiety Disorders.

[B21-behavsci-16-01801] Clayton S., Czellar S., Nartova-Bochaver S., Skibins J. C., Salazar G., Tseng Y.-C., Irkhin B., Monge-Rodriguez F. S. (2021). Cross-cultural validation of A revised environmental identity scale. Sustainability.

[B22-behavsci-16-01801] Clayton S., Karazsia B. T. (2020). Development and validation of a measure of climate change anxiety. Journal of Environmental Psychology.

[B23-behavsci-16-01801] Clayton S., Pihkala P., Wray B., Marks E. (2023). Psychological and emotional responses to climate change among young people worldwide: Differences associated with gender, age, and country. Sustainability.

[B24-behavsci-16-01801] Cloitre M., Garvert D. W., Brewin C. R., Bryant R. A., Maercker A. (2013). Evidence for proposed ICD-11 PTSD and complex PTSD: A latent profile analysis. European Journal of Psychotraumatology.

[B25-behavsci-16-01801] Cloitre M., Hyland P., Bisson J. I., Brewin C. R., Roberts N. P., Karatzias T., Shevlin M. (2019). ICD-11 posttraumatic stress disorder and complex posttraumatic stress disorder in the United States: A population-based study: PTSD and complex PTSD in the United States. Journal of Traumatic Stress.

[B26-behavsci-16-01801] Cloitre M., Shevlin M., Brewin C. R., Bisson J. I., Roberts N. P., Maercker A., Karatzias T., Hyland P. (2018). The international trauma questionnaire: Development of a self-report measure of ICD-11 PTSD and complex PTSD. Acta Psychiatrica Scandinavica.

[B27-behavsci-16-01801] Collins N. L. (1996). Working models of attachment: Implications for explanation, emotion, and behavior. Journal of Personality and Social Psychology.

[B28-behavsci-16-01801] Collins N. L., Read S. J. (1990). Adult attachment, working models, and relationship quality in dating couples. Journal of Personality and Social Psychology.

[B29-behavsci-16-01801] Cox D. R., Snell E. J. (1989). The analysis of binary data.

[B30-behavsci-16-01801] Çimen F., Seçer İ., Pakiş A. (2025). Assessment of PTSD and CPTSD symptoms in children and adolescents exposed to earthquakes: An evaluation using latent profile. Clinical Psychology & Psychotherapy.

[B31-behavsci-16-01801] Dias A., Carvalho A., Castro-Vale I., Sales L., Cardoso R. M. (2013). Estudo de propriedades psicométricas do Questionário de Trauma de Infância—Versão breve numa amostra portuguesa não clínica. Laboratório de Psicologia.

[B32-behavsci-16-01801] Doherty T. J., Clayton S. (2011). The psychological impacts of global climate change. American Psychologist.

[B33-behavsci-16-01801] Ferrajão P., Torres N., Martins A. Q. (2024). Adaptation of the revised environmental identity scale to adult Portuguese native speakers: A validity and reliability study. Sustainability.

[B34-behavsci-16-01801] Ferrajão P., Torres N., Martins A. Q. (2026). Latent profiles of eco-anxiety: Resilience, and vulnerability factors in a Portuguese-sample. Sustainability.

[B35-behavsci-16-01801] Ferrajão P., Torres N., Quelhas Martins A. (2025). Adaptation of the eco-anxiety scale to adult Portuguese native speakers: A validity and reliability study. SAGE Open.

[B36-behavsci-16-01801] Folke S., Nielsen A. B. S., Andersen S. B., Karatzias T., Karstoft K.-I. (2019). ICD-11 PTSD and complex PTSD in treatment-seeking Danish veterans: A latent profile analysis. European Journal of Psychotraumatology.

[B37-behavsci-16-01801] Gauthier G. M., Pandey S., Sanchez V., Stromberg S. E., Zoellner L. A. (2025). Posttraumatic stress disorder and climate change: A meta-analysis of prospective, acute predictors of PTSD following extreme weather events. Clinical Psychology Review.

[B38-behavsci-16-01801] Hair J. F., Black W. C., Babin B. J., Anderson R. E. (2010). Multivariate data analysis.

[B39-behavsci-16-01801] Hayes K., Blashki G., Wiseman J., Burke S., Reifels L. (2018). Climate change and mental health: Risks, impacts and priority actions. International Journal of Mental Health Systems.

[B40-behavsci-16-01801] Herman J. L. (2015). Trauma and recovery: The aftermath of violence—From domestic abuse to political terror.

[B41-behavsci-16-01801] Hickman C. (2020). We need to (find a way to) talk about … eco-anxiety. Journal of Social Work Practice.

[B42-behavsci-16-01801] Hogg T. L., Stanley S. K., O’Brien L. V., Wilson M. S., Watsford C. R. (2021). The Hogg eco-anxiety scale: Development and validation of a multidimensional scale. Global Environmental Change: Human and Policy Dimensions.

[B43-behavsci-16-01801] Howard M. C., Hoffman M. E. (2018). Variable-centered, person-centered, and person-specific approaches: Where theory meets the method. Organizational Research Methods.

[B44-behavsci-16-01801] Hualparuca-Olivera L., Vigo-Ayasta E., Torales J., Ramos-Vera C., Caycho-Rodríguez T., Calle-Arancibia M., Calle D., Ventriglio A. (2026). A systematic review and meta-analysis of the global prevalence of ICD-11 complex post-traumatic stress disorder in the community. The International Journal of Social Psychiatry.

[B45-behavsci-16-01801] Hyland P., Murphy J., Shevlin M., Vallières F., McElroy E., Elklit A., Christoffersen M., Cloitre M. (2017). Variation in post-traumatic response: The role of trauma type in predicting ICD-11 PTSD and CPTSD symptoms. Social Psychiatry and Psychiatric Epidemiology.

[B46-behavsci-16-01801] Jalin H., Sapin A., Macherey A., Boudoukha A. H., Congard A. (2024). Understanding eco-anxiety: Exploring relationships with environmental trait affects, connectedness to nature, depression, anxiety, and media exposure. Current Psychology.

[B47-behavsci-16-01801] Jennings V., Bamkole O. (2019). The relationship between social cohesion and urban green space: An avenue for health promotion. International Journal of Environmental Research and Public Health.

[B48-behavsci-16-01801] Johnson S. T., Mason S. M., Erickson D., Slaughter-Acey J. C., Waters M. C. (2024). Predicting post-disaster post-traumatic stress disorder symptom trajectories: The role of pre-disaster traumatic experiences. International Journal of Environmental Research and Public Health.

[B49-behavsci-16-01801] Kairyte A., Kvedaraite M., Kazlauskas E., Gelezelyte O. (2022). Exploring the links between various traumatic experiences and ICD-11 PTSD and complex PTSD: A cross-sectional study. Frontiers in Psychology.

[B50-behavsci-16-01801] Kaplan E. A. (2020). Is climate-related pre-traumatic stress syndrome a real condition?. American Imago.

[B51-behavsci-16-01801] Karatzias T., Hyland P., Bradley A., Cloitre M., Roberts N. P., Bisson J. I., Shevlin M. (2019). Risk factors and comorbidity of ICD-11 PTSD and complex PTSD: Findings from a trauma-exposed population based sample of adults in the United Kingdom. Depression and Anxiety.

[B52-behavsci-16-01801] Kline R. B. (2016). Principles and practice of structural equation modeling.

[B53-behavsci-16-01801] Koder J., Dunk J., Rhodes P. (2023). Climate distress: A review of current psychological research and practice. Sustainability.

[B54-behavsci-16-01801] Lanza S. T., Tan X., Bray B. C. (2013). Latent class analysis with distal outcomes: A flexible model-based approach. Structural Equation Modeling.

[B55-behavsci-16-01801] Li D., Luo J., Yan X., Liang Y. (2023). Complex posttraumatic stress disorder (CPTSD) as an independent diagnosis: Differences in hedonic and eudaimonic well-being between CPTSD and PTSD. Healthcare.

[B56-behavsci-16-01801] Liang Y., Zhao Y., Ji W., Bian X., Xi J. (2023). Latent profiles of psychological status among populations cumulatively exposed to a flood and the recurrence of the COVID-19 pandemic in China. International Journal of Disaster Risk Reduction 85.

[B57-behavsci-16-01801] Liu A., Wang W., Wu X. (2021). Self-compassion and posttraumatic growth mediate the relations between social support, prosocial behavior, and antisocial behavior among adolescents after the Ya’an earthquake. European Journal of Psychotraumatology.

[B58-behavsci-16-01801] Lo Y., Mendell N. R., Rubin D. B. (2001). Testing the number of components in a normal mixture. Biometrika.

[B59-behavsci-16-01801] Lowe S. R., Dillon C. O., Rhodes J. E., Zwiebach L. (2013). Defining adult experiences: Perspectives of a diverse sample of young adults. Journal of Adolescent Research.

[B60-behavsci-16-01801] Lutz P. K., Zelenski J. M., Newman D. B. (2023). Eco-anxiety in daily life: Relationships with well-being and pro-environmental behavior. Current Research in Ecological and Social Psychology.

[B61-behavsci-16-01801] Martin L., White M. P., Hunt A., Richardson M., Pahl S., Burt J. (2020). Nature contact, nature connectedness and associations with health, wellbeing and pro-environmental behaviours. Journal of Environmental Psychology.

[B62-behavsci-16-01801] Masyn K. E., Little T. D. (2013). Latent class analysis and finite mixture modeling. The Oxford handbook of quantitative methods: Statistical analysis.

[B63-behavsci-16-01801] Morison M., Benight C. C. (2022). Trauma coping self-efficacy mediates associations between adult attachment and posttraumatic stress symptoms. Frontiers in Psychology.

[B64-behavsci-16-01801] Nagelkerke N. J. D. (1991). A note on a general definition of the coefficient of determination. Biometrika.

[B65-behavsci-16-01801] Nan J., Jaiswal S., Ramanathan D., Withers M. C., Mishra J. (2025). Climate trauma from wildfire exposure impacts cognitive decision-making. Scientific Reports.

[B66-behavsci-16-01801] O’Donnell M., Palinkas L. (2024). Taking a trauma and adversity perspective to climate change mental health. European Journal of Psychotraumatology.

[B67-behavsci-16-01801] Oh R. R. Y., Zhang Y., Nghiem L. T. P., Chang C.-C., Tan C. L. Y., Quazi S. A., Shanahan D. F., Lin B. B., Gaston K. J., Fuller R. A., Carrasco R. L. (2022). Connection to nature and time spent in gardens predicts social cohesion. Urban Forestry & Urban Greening.

[B68-behavsci-16-01801] Ojala M., Cunsolo A., Ogunbode C., Middleton J. (2021). Anxiety, worry, and grief in a time of environmental and climate crisis: A narrative review. Annual Review of Environment and Resources.

[B69-behavsci-16-01801] Palinkas L. A., Wong M. (2020). Global climate change and mental health. Current Opinion in Psychology.

[B70-behavsci-16-01801] Patrick R., Snell T., Gunasiri H., Garad R., Meadows G., Enticott J. (2023). Prevalence and determinants of mental health related to climate change in Australia. The Australian and New Zealand Journal of Psychiatry.

[B71-behavsci-16-01801] Pedretti B., Lavazza A. (2026). Climate-related flooding and mental health vulnerability: Post-traumatic stress, psychological distress, and climate worry in a European flood-affected population. Environmental Challenges.

[B72-behavsci-16-01801] Pihkala P. (2020). Anxiety and the ecological crisis: An analysis of eco-anxiety and climate anxiety. Sustainability.

[B73-behavsci-16-01801] Redican E., Cloitre M., Hyland P., McBride O., Karatzias T., Murphy J., Shevlin M. (2022). The latent structure of ICD-11 posttraumatic stress disorder (PTSD) and complex PTSD in a general population sample from USA: A factor mixture modelling approach. Journal of Anxiety Disorders.

[B74-behavsci-16-01801] Rocha J., Rodrigues V., Santos E., Azevedo I., Machado S., Almeida V., Silva C., Almeida J., Cloitre M. (2022). Primeiro instrumento para avaliação de TEPT complexo: Propriedades psicométricas do International Trauma Questionnaire da CID-11. Debates em Psiquiatria.

[B75-behavsci-16-01801] Rossi R., Socci V., Pacitti F., Carmassi C., Rossi A., Di Lorenzo G., Hyland P. (2022). The Italian version of the international trauma questionnaire: Symptom and network structure of post-traumatic stress disorder and complex post-traumatic stress disorder in a sample of late adolescents exposed to a natural disaster. Frontiers in Psychiatry.

[B76-behavsci-16-01801] Scheffran J. (2025). Systemic risks and governance of the global polycrisis in the Anthropocene: Stability of the climate–conflict–migration–pandemic nexus. Global Sustainability.

[B77-behavsci-16-01801] Shaligram D., Acquaye A., Wortzel J. R., DeJong S. M. (2025). Climate mental health disparities: A biopsychosociocultural perspective. Journal of the American Academy of Child and Adolescent Psychiatry.

[B78-behavsci-16-01801] Shomuyiwa D. O., Lucero-Prisno D. E. (2025). Climate change trauma and collective dissociation: Unraveling the impact on mental health and advocating for collective action. Global Mental Health.

[B79-behavsci-16-01801] Smith C., Allen A., Schaffer V., Kannis-Dymand L. (2024). Nature relatedness may play a protective role and contribute to eco-distress. Ecopsychology.

[B80-behavsci-16-01801] Stanley S. K., Hogg T. L., Leviston Z., Walker I. (2021). From anger to action: Differential impacts of eco-anxiety, eco-depression, and eco-anger on climate action and wellbeing. The Journal of Climate Change and Health.

[B81-behavsci-16-01801] Tang B., Deng Q., Glik D., Dong J., Zhang L. (2017). A meta-analysis of risk factors for post-traumatic stress disorder (PTSD) in adults and children after earthquakes. International Journal of Environmental Research and Public Health.

[B82-behavsci-16-01801] Tekin S., Burrows K., Billings J., Waters M., Lowe S. R. (2023). Psychosocial resources underlying disaster survivors’ posttraumatic stress symptom trajectories: Insight from in-depth interviews with mothers who survived Hurricane Katrina. European Journal of Psychotraumatology.

[B83-behavsci-16-01801] Teysseyre J., Tapia G., Raysséguier C., Chami L., Gissot V., El-Hage W. (2026). Latent profiles of PTSD and CPTSD: The importance of emotional hypoactivation. European Journal of Psychotraumatology.

[B84-behavsci-16-01801] Thoma M. V., Redican E., Adank N., Schneemann V. B., Shevlin M., Maercker A., Rohner S. L. (2025). ICD-11 posttraumatic stress disorder and complex PTSD: Prevalence, predictors, and construct validity in Swiss older adults. European Journal of Psychotraumatology.

[B85-behavsci-16-01801] Thoma M. V., Rohleder N., Rohner S. L. (2021). Clinical ecopsychology: The mental health impacts and underlying pathways of the climate and environmental crisis. Frontiers in Psychiatry.

[B86-behavsci-16-01801] Thomson E. E., Roach S. P. (2023). The relationships among nature connectedness, climate anxiety, climate action, climate knowledge, and mental health. Frontiers in Psychology.

[B87-behavsci-16-01801] Ungar M., Theron L. (2020). Resilience and mental health: How multisystemic processes contribute to positive outcomes. The Lancet Psychiatry.

[B88-behavsci-16-01801] Wang S., van den Berg B. (2021). Neuroscience and climate change: How brain recordings can help us understand human responses to climate change. Current Opinion in Psychology.

[B89-behavsci-16-01801] White M. P., Elliott L. R., Grellier J., Economou T., Bell S., Bratman G. N., Cirach M., Gascon M., Lima M. L., Lõhmus M., Nieuwenhuijsen M., Ojala A., Roiko A., Schultz P. W., van den Bosch M., Fleming L. E. (2021). Associations between green/blue spaces and mental health across 18 countries. Scientific Reports.

[B90-behavsci-16-01801] Woodbury Z. (2019). Climate trauma: Toward a new taxonomy of trauma. Ecopsychology.

[B91-behavsci-16-01801] Woodhouse S., Ayers S., Field A. P. (2015). The relationship between adult attachment style and post-traumatic stress symptoms: A meta-analysis. Journal of Anxiety Disorders.

[B92-behavsci-16-01801] World Health Organization (2022). ICD-11: International classification of diseases (11th revision).

[B93-behavsci-16-01801] Yakubu S., Mousavi S., Palade V., Eden J., Kabajulizi J., Salari N., Hosseinian-Far A., Al Ali H., Daneshkhah A. (2026). Flooding and the risk of PTSD, depression, and anxiety: A systematic review and meta-analysis. Environmental Research.

[B94-behavsci-16-01801] Zhang S., Braithwaite I., Bhavsar V., Das-Munshi J. (2021). Unequal effects of climate change and pre-existing inequalities on the mental health of global populations. BJPsych Bulletin.

[B95-behavsci-16-01801] Zhen R., Quan L., Zhou X. (2020). Co-occurring patterns of post-traumatic stress disorder and depression among flood victims: A latent profile analysis. Journal of Health Psychology.

